# Functional Characterization of Nuclear Localization and Export Signals in Hepatitis C Virus Proteins and Their Role in the Membranous Web

**DOI:** 10.1371/journal.pone.0114629

**Published:** 2014-12-08

**Authors:** Aviad Levin, Christopher J. Neufeldt, Daniel Pang, Kristen Wilson, Darci Loewen-Dobler, Michael A. Joyce, Richard W. Wozniak, D. Lorne J Tyrrell

**Affiliations:** 1 Li Ka Shing Institute of Virology, Department of Medical Microbiology and Immunology, University of Alberta, Edmonton, Alberta, Canada; 2 Department of Cell Biology, University of Alberta, Edmonton, Alberta, Canada; UMR Inserm U1052 / CNRS 5286, France

## Abstract

The hepatitis C virus (HCV) is a positive strand RNA virus of the *Flavivirus* family that replicates in the cytoplasm of infected hepatocytes. Previously, several nuclear localization signals (NLS) and nuclear export signals (NES) have been identified in HCV proteins, however, there is little evidence that these proteins travel into the nucleus during infection. We have recently shown that nuclear pore complex (NPC) proteins (termed nucleoporins or Nups) are present in the membranous web and are required during HCV infection. In this study, we identify a total of 11 NLS and NES sequences in various HCV proteins. We show direct interactions between HCV proteins and importin α5 (IPOA5/kapα1), importin β3 (IPO5/kap β3), and exportin 1 (XPO1/CRM1) both *in-vitro* and in cell culture. These interactions can be disrupted using peptides containing the specific NLS or NES sequences of HCV proteins. Moreover, using a synchronized infection system, we show that these peptides inhibit HCV infection during distinct phases of the HCV life cycle. The inhibitory effects of these peptides place them in two groups. The first group binds IPOA5 and inhibits infection during the replication stage of HCV life cycle. The second group binds IPO5 and is active during both early replication and early assembly. This work delineates the entire life cycle of HCV and the active involvement of NLS sequences during HCV replication and assembly. Given the abundance of NLS sequences within HCV proteins, our previous finding that Nups play a role in HCV infection, and the relocation of the NLS double-GFP reporter in HCV infected cells, this work supports our previous hypothesis that NPC-like structures and nuclear transport factors function in the membranous web to create an environment conducive to viral replication.

## Introduction

Hepatitis C virus (HCV) is a positive strand RNA virus of the *Flaviviradae* family [1], [2], a blood borne pathogen and a major cause of liver disease, with an estimated 170 million people infected worldwide [3]. It is estimated that approximately 30% of HCV-infected patients will develop cirrhosis [1], [4].

The current model of HCV entry involves the binding of HCV to a variety of receptors including glycoaminoglycans [5], the LDL receptor [6], [7], CD81 [8], and SR-B1 [9], [10] followed by clathrin-dependent internalization at tight junctions [11]–[13], additional receptors used by HCV for entry include Claudin-1 [14], Occludin [12], EGFR and EphA2 [15]. Following fusion of the viral envelope with the membrane of acidified endosomes [13], [16], the viral genomic RNA is released into the cytoplasm and translated on the rough endoplasmic reticulum (ER) [13]. After polyprotein cleavage, viral proteins modify host membranes to generate the membranous web, the site of viral replication and assembly [17]–[20]. Virus assembly occurs in association with lipid droplets [21], and HCV appears to utilize the very-low-density lipoprotein (VLDL) secretion machinery for viral egress from the cell [22]–[25].

Plus strand RNA viruses induce the rearrangement of host cell membranes, including the ER, Golgi complex, mitochondria, endosomes, peroxisomes and others, to facilitate viral replication and assembly [18], [26]–[28]. HCV infection leads to extensive re-organization of host cell membranes into the membranous web, which arises primarily from the ER. The membranous web has been shown to protect the viral genome from exogenously added nucleases [18], [29], [30].

It has been proposed that the membranous web constitutes a virally-encoded organelle within the host cell cytoplasm [29], [31]. Consistent with this idea, we have shown that the membranous web occupies regions of the cytoplasm that are definable by using antibodies directed against HCV proteins and excluding microtubules [32]. The membranous web has been proposed to concentrate and synchronize virus replication, assembly, and egress. Moreover, the membranous web may restrict access of host cytoplasmic pattern recognition receptors (PRR) to the replicating virus [26], [33]. All of these functions necessitate the existence of a selective permeable barrier between the interior of the membranous web and the surrounding cytosol.

Nuclear pore complexes (NPCs) are large macromolecular structures positioned in the nuclear envelope that form a permeability barrier between the cytoplasm and the nucleoplasm. Small molecules such as metabolites can freely diffuse though the NPCs however, most proteins and protein-nucleic acid complexes require a short sequence of amino acid residues, termed a nuclear localization signal (NLS) or nuclear export signal (NES), to enter or leave the nucleus through NPCs [34], [35]. There are multiple NLSs and NESs and, in turn, multiple nuclear transport factors (NTFs) that bind these signals. The first identified “classical NLS”, typically contains arginine-rich motifs and is recognized by an importin α (IPOA/Kap α)/importin β (IPO/Kap β) complex in the cytoplasm that is transported through the NPC. Within the nucleus importins are dissociated from the NLS-containing cargo. The importins are then recycled back to the cytoplasm [36]. By contrast, nuclear export factors, such as XPO1, bind NES sequences of export cargos in the nucleoplasm in a stable trimeric complex containing RanGTP. Upon entry to the cytoplasm, RanGTP is converted to RanGDP and the complex is dissociated [34], [35].

Some flaviviruses have been reported to replicate in the nucleus [37], [38], and there are several reports that HCV proteins and proteins of other flaviviruses contain nuclear NLS and NES [39]–[41]. In addition, specific NPC proteins (Nups) have been shown to interact with viral proteins during infection with several viruses such as influenza [42], hepatitis B virus [43], poliovirus [44], vesicular stomatitis virus [45], dengue virus [46] and HCV [47]. Recently, we have shown the involvement of Nups in the HCV life cycle and propose that NPC-like structures constitute a selective permeability barrier in the membranous web of HCV-infected cells [32].

The HCV life cycle occurs in the cytoplasm and has no clear nuclear intermediate step. Therefore, the function of viral protein interactions with NTFs or Nups is unknown. Nevertheless, the multitude of interactions that have been reported between HCV proteins and NTFs or Nups suggests that such interactions play a significant role in the viral life cycle. Three of the ten HCV proteins (core, NS3 and NS5A) have been reported to contain putative NLS sequences, and these proteins, in either truncated or mutated forms, can enter the nucleus when produced outside of the context of viral infection. However, they have not been detected in the nuclei of HCV-infected hepatocytes [39], [40], [48]–[51]. In addition to the NLSs, core was also recently reported to have a functional NES [39]. The functionality of these transport signals is also supported by recent co-immunoprecipitation (Co-IP) studies in which various HCV proteins have been detected in association with NTFs including IPO5, IPO7, IPO8, IPO9, XPO1, XPO5, XPOT, and transportin 1 (TNPO1) [52].

In this study, we identify and characterize nuclear transport signals, namely, NLSs and NESs, within several HCV proteins. We demonstrate that these signals bind specific NTFs both *in-vitro* and in cell culture and are capable of supporting nuclear transport of reporter proteins. We present evidence that the various HCV nuclear transport signals function at specific times during HCV infection. For example, early acting peptides bearing NLSs which bind to IPOA5, reduce the production of both intracellular and extracellular viral RNA to a similar extent, and have the ability to direct reporter proteins to both the nucleus and the membranous web of HCV infected cells. On the other hand, peptides bearing NLS sequences that bind to IPO5 have additional effects late in infection and are excluded from the membranous web. We propose that the recruitment of NPCs to the membranous web allows nuclear transport proteins to regulate access of host and viral proteins to the membranous web.

## Results

### Identification of potential nuclear transport signals within the HCV protein

**Figure 1:**
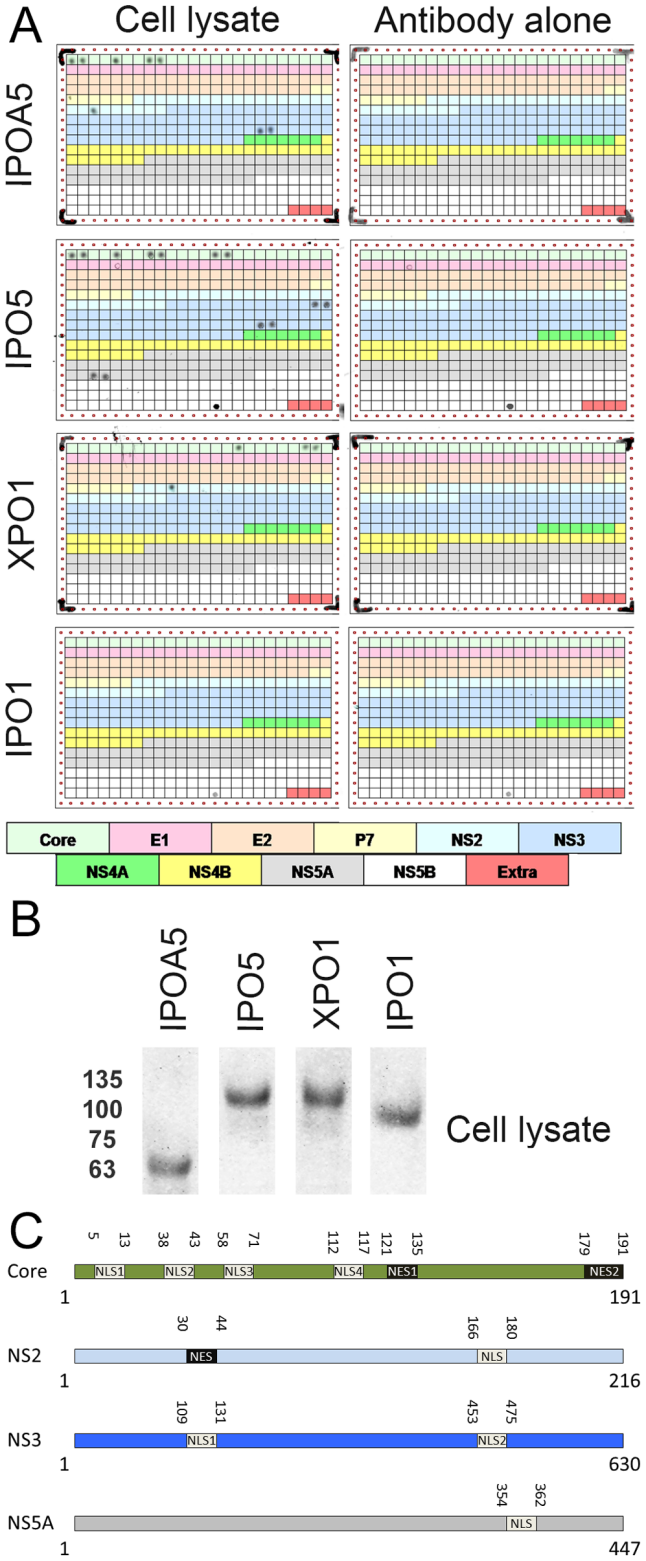
Identification of sequences within the HCV polyprotein that bind nuclear transport factors. (A) HCV genotype 1 peptide arrays consisting of 15 residue peptides (with 8 residue overlaps) spanning the complete HCV polypeptide were incubated with Huh7.5 cells lysates. Bound NTFs were detected using antibodies specific for the indicated NTF. Control peptide arrays were incubated with only NTF-specific antibodies. The different colored regions of the array represent peptides derived from different HCV proteins (see schematic). The last four peptides on the array are additional sequences which some of the HCV strains have in their polyproteins at the C terminus. The peptide sequences that bound to NTFs are summarized in Table 1. (B) The same lysate used to probe the peptide arrays was also analyzed by western blotting to detect the indicated NTFs and assess the specificities of the antibodies. (C) Schematic summary of the localization of the NTF binding sites on the indicated HCV proteins.

HCV does not have a defined nuclear step in its life cycle. However, various HCV proteins contain putative NLSs and NESs [39], [41], [53]. To identify potential interactions between cellular NTFs and HCV proteins, we designed a peptide array spanning the entire HCV genotype 1 consensus polyprotein sequence. These arrays were incubated with or without cell lysates and then probed using specific antibodies for components of the nuclear transport machinery. We detected binding of IPOA5 (importin α5/kap α1), IPO5 (importin β3/kap β3) and XPO1 (CRM1), but not IPO1 (importin β1/kap β1), to eleven distinct regions within HCV proteins (Fig. 1A, Table 1). All of the antibodies were also tested by western blotting of cell lysates to validate that they detected the corresponding NTF (Fig. 1B). Among the binding sites detected on the array, six regions within core and one in NS5A correspond to domains previously suggested to function as NLSs or NESs [39], [54]. In addition, two putative NLSs in NS3 and one in NS2 were identified (Fig. 1A, Table 1). These NTF binding sites exhibit different levels of specificity for the various NTFs. Three of four potential NLSs in core and one in NS3 bound both IPOA5 and IPO5. By contrast, the potential NLS in NS2 bound only to IPOA5, while the fourth NLS of core, the first potential NLS domain in NS3, and the NLS in NS5A bound exclusively to IPO5 (Fig. 1A, and Table 1). We also detected binding of XPO1 to two previously reported NES sequences in core [39], and we detected an additional XPO1 binding site in NS2 (Fig. 1A, Table 1). A diagram summarizing the position of the potential nuclear transport signals in the HCV proteins is presented in Fig. 1C. All the putative nuclear transport signals identified in the HCV proteins were highly conserved between the different genotypes, suggesting their functions are conserved in all genotypes of HCV.

**Table 1:** Summary of the peptides selected by peptide array analysis.

Protein	Binding to IPOA5	Binds to IPO5	Binding to XPO1
**Core**	A1: 1-MSTNPKPQRKTKRNT-15	A1: 1-MSTNPKPQRKTKRNT-15	
	A2: 9-RKTKRNTNRRPEDVK-23	A2: 9-RKTKRNTNRRPEDVK-23	
	A5: 33-GVYLLPRRGPRLGVR-47	A5: 33-GVYLLPRRGPRLGVR-47	
	A8: 57-QPRGRRQPIPKDRRS-71	A8: 57-QPRGRRQPIPKDRRS-71	
	A9: 65-IPKDRRSTGKAWGKP-79	A9: 65-IPKDRRSTGKAWGKP-79	
		A14: 105-PSWGPTDPRHRSRNV-119	
		A15: 113-RHRSRNVGKVIDTLT-127	
			A16: 121-KVIDTLTCGFADLMG-135
			A22: 169-LPGFPFSIFLLALLS-183
			A23: 177-FLLALLSCITVPVSA-191
**NS2**			E10: 841-TLLGQCLWWLCYLLT-855
	F3: 977-PIIFSPMEKKVIVWG-991		
**NS3**		F23: 1137-VTRNADVIPARRRGD-1151	
		F24: 1145-PARRRGDKRGALLSP-1159	
	H18: 1481-VPQDAVSRSQRRGRT-1495	H18: 1481-VPQDAVSRSQRRGRT-1495	
	H19: 1489-SQRRGRTGRGRQGTY-1503	H19: 1489-SQRRGRTGRGRQGTY-1503	
**NS5A**		M3: 2321-KAPTPPPRRRRTVGL-2335	
		M4: 2329-RRRTVGLSESTISEA-2343	

To further characterize the qualitative interactions described above, we determined the dissociation constants (K_d(app)_) between synthetic peptides containing the potential HCV nuclear transport signals fused to a penetratin sequence, as well as the penetratin peptide (Pen) [55] (Table 2) and IPOA5, IPO5, or XPO1 by ELISA. The penetratin sequence is a cell permeable peptide [55] that was fused to the HCV sequences to facilitate entry into tissue culture cells. Immobilized nuclear transport signals bearing peptides were incubated with increasing concentrations of recombinant IPOA5, IPO5, or XPO1. Binding was detected using specific antibodies directed against the NTFs. The binding curves are presented in S1A-F Figure and the calculated K_d(app)_ are presented in Table 3A. The relative affinities of the importins for the peptides were consistent with the results obtained using the array. The NLS peptide found in NS2 bound only to IPOA5 (K_d(app)_ ∼34 nM). Similarly, NLS 1 in NS3 bound only to IPO5 (K_d(app)_ ∼33 nM), while the NLS in NS5A and core NLS 4 had greater affinities for IPO5 (K_d(app)_ ∼32 nM and ∼105 nM, respectively) than for IPOA5 (K_d(app)_ ∼7.6 µM and ∼0.52 µM, respectively). The affinities of the importins for the HCV NLS peptides were similar to those measured for IPOA5 binding to SV40 NLS (K_d(app)_ ∼27 nM) and IPO5 binding to HIV-1 Rev NLS (K_d(app)_ ∼44 nM) (S1F Figure and Table 3A); the latter is comparable to published values [56], [57]. Peptides with the same amino acid residue sequence, but in reverse order (SLN) did not bind either importin. All of the potential NES domains identified in the array bound to XPO1 with an affinity similar to that of the NES from the HIV-1 REV protein (S1E Figure, Table 3A; [56]). Again, peptides with the reverse sequence (SEN) did not bind XPO1. These findings support the peptide array results, and show that the nuclear transport signals identified in HCV proteins exhibit affinities for NTFs similar to those previously reported for other nuclear transport signals/NTF interactions [56], [57].

**Table 2:** Synthetic peptides.

Name	Sequence
Core NLS 1	**PKPQRKTKRNTNRRP***RQIKIWFQNRRMKWKK*
Core NLS 2	**PRRGPRLGVR***RQIKIWFQNRRMKWKK*
Core NLS 3	**PRGRRQPIPKDRRSTGKAWGKP***RQIKIWFQNRRMKWKK*
Core NLS 4	**PRHRSRNVGK***RQIKIWFQNRRMKWKK*
NS2 NLS	**PIIFSPMEKKVIVWG***RQIKIWFQNRRMKWKK*
NS3 NLS 1	**PARRRGDKR***RQIKIWFQNRRMKWKK*
NS3 NLS 2	**RSQRRGRTTGRGR***RQIKIWFQNRRMKWKK*
NS5A NLS	**PPPRRRRTV***RQIKIWFQNRRMKWKK*
HIV-1 Rev NLS	**TRQARRNRRRRWRERQR**
SV40 NLS	**PKKKRKV***RQIKIWFQNRRMKWKK*
SV40 mut NLS	**PKTKRKV***RQIKIWFQNRRMKWKK*
Core NES 1	**KVIDTLTCGFADLMG***RQIKIWFQNRRMKWKK*
Core NES 2	**LPGCSFSIFLLALLSCITVPVSA***RQIKIWFQNRRMKWKK*
NS2 NES	**LILLTLSPHYKLFLARLIWWLQYFIT***RQIKIWFQNRRMKWKK*
HIV-1 Rev NES	**LQLPPLERLTL***RQIKIWFQNRRMKWKK*
Core SLN 1	**PRRNTNRKTKRQPKP***RQIKIWFQNRRMKWKK*
Core SLN 2	**RVGLRPGRRP***RQIKIWFQNRRMKWKK*
Core SLN 3	**PKGWAKGTSRRDKPIPQRRGRP***RQIKIWFQNRRMKWKK*
Core SLN 4	**KGVNRSRHRP***RQIKIWFQNRRMKWKK*
NS2 SLN	**GWVIVKKEMPSFIIP***RQIKIWFQNRRMKWKK*
NS3 SLN 1	**RKDGRRRAP***RQIKIWFQNRRMKWKK*
NS3 SLN 2	**RGRGTTRGRRQSR***RQIKIWFQNRRMKWKK*
NS5A SLN	**VTRRRRPPP***RQIKIWFQNRRMKWKK*
HIV-1 Rev SLN	**RQRERWRRRRNRRAQRT***RQIKIWFQNRRMKWKK*
SV40 SLN	**VKRKKKP***RQIKIWFQNRRMKWKK*
Core SEN 1	**GMLDAFGCTLTDIVK***RQIKIWFQNRRMKWKK*
Core SEN 2	**ASVPVTICSLLALLFISFSCGPL***RQIKIWFQNRRMKWKK*
NS2 SEN	**TIFYQLWWILRALFLKYHPSLTLLIL***RQIKIWFQNRRMKWKK*
HIV-1 Rev SEN	**LTLRELPPLQL***RQIKIWFQNRRMKWKK*
Pen	*RQIKIWFQNRRMKWKK*

In italic the penetratin (pen) sequence and in bold the nuclear transport signal or the reverse signal.

**Table 3:** Binding affinity of NTFs to nuclear transport signals and to HCV proteins.

	Peptide	K_d(app)_ [nM]		
		IPOA5	IPO5	XPO1
A	Core NLS 1	161.3±31.8	34.75±4.54	-
	Core NLS 2	24.88±1.71	78.84±6.42	-
	Core NLS 3	17.47±0.81	40.44±7.06	-
	Core NLS 4	517.8±28.6	104.7±31.1	-
	NS2 NLS	79.25±3.89	ND	-
	NS3 NLS 1	ND	33.83±5.60	-
	NS3 NLS 2	36.56±7.13	35.78±7.26	-
	NS5A NLS	7632±258.64	32.29±4.986	-
	HIV-1 Rev NLS	ND	44.92±6.75	-
	SV40 NLS	27.15±4.23	ND	-
	SV40 mut NLS	ND	-	-
	Core NES 1	-	-	66.73±11.30
	Core NES 2	-	-	95.24±26.58
	NS2 NES	-	-	79.85±25.62
	HIV-1 Rev NES	-	-	114.30±71.53
B	**Protein**			
	Core	36.80±4.83	56.05±2.78	23.08±4.07
	NS2	37.10±5.76	ND	51.93±11.59
	NS3	48.34±6.23	41.90±3.88	ND
	NS5A	ND	35.61±1.38	ND

ND – Not detected.

The presence of NTF binding domains in the various HCV proteins suggested that the full-length proteins were capable of binding NTFs; however, for this binding to occur, the NTF binding domain must be exposed. To directly test this, we examined the binding of full-length HCV proteins to IPOA5, IPO5, and XPO1. For these studies, affinity-purified recombinant HCV proteins were used in ELISA assays to assess their binding to NTFs (S1G-I Figure, Table 3B). Consistent with the peptide binding results, core, NS2, and NS3 bound IPOA5 with K_d(app)_ values in the low nanomolar range (36–48 nM); however, NS5A did not bind IPOA5 (S1G Figure, Table 3B). Core, NS3 and NS5A bound IPO5 (K_d(app)_ values ranging from 35–56 nM), while NS2 did not bind IPO5 (S1H Figure, Table 3B). Finally, the two proteins bearing the potential NES peptides, core and NS2, bound to XPO1 (K_d(app)_ ∼23 nM and 52 nM respectively) (S1I Figure, Table 3B). As expected, those viral proteins that did not contain a consensus NES, namely, NS3 and NS5A, did not bind to XPO1 (S1I Figure, Table 3B). In summary, these results are consistent with the peptide binding data and support the conclusion that core and NS3 can bind two importins, IPOA5 and IPO5, while NS5A and NS2 bind a specific NTF (IPO5 or IPOA5, respectively).

**Figure 2:**
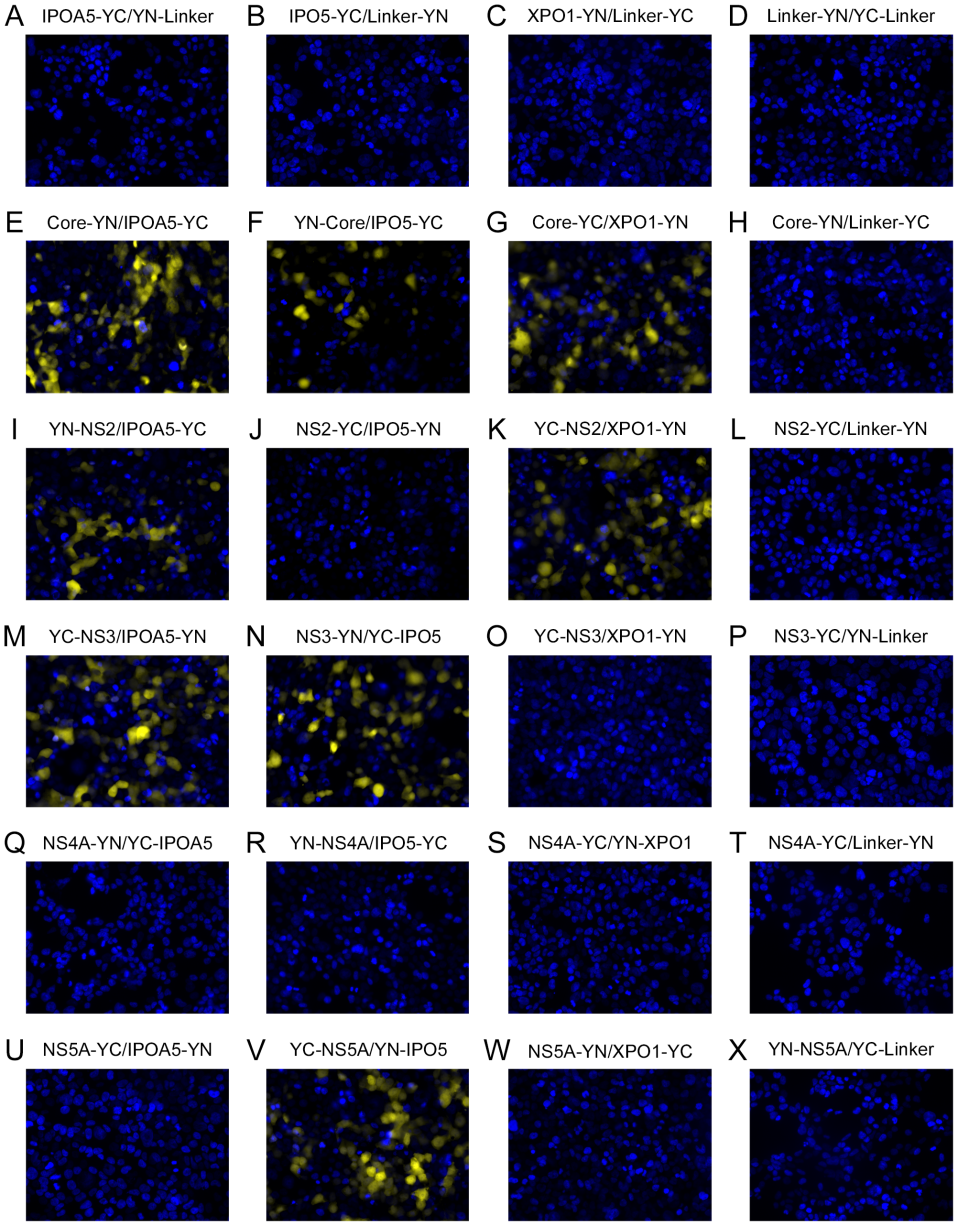
Binding of HCV proteins to nuclear transport factors in transfected cells using BiFC assays. 293T cells were co-transfected with plasmids encoding the indicated HCV protein conjugated to half of a YFP molecule and NTFs conjugated to the complementary half of the YFP molecule. At 36 h post transfection, cells were fixed and stained with Hoechst. Cells were examined using a Zeiss inverted Axiovert 200 M microscope to visualize Hoechst (blue) and YFP (yellow). This figure shows one of the 8 BiFC combinations for each pair of proteins tested for their interaction. The additional BiFC results are summarized in S1 Table.

We examined whether these specific interactions could be detected in cell culture using the bimolecular fluorescence complementation (BiFC) system (Fig. 2) [58], [59]. BiFC is capable of detecting protein-protein interactions in cells by monitoring the formation of a fluorescent protein, e.g., YFP, from two halves of YFP separately expressed as a fusion with each of two potentially interacting partners. When one or both halves of YFP alone are produced in cells (Fig. 2A-D) or as chimeras with non-interacting proteins, no fluorescent signal is detected. However, if the chimeras interact, the potential exists for the two halves of the YFP to be placed in a confirmation that allows formation of the fluorophore. We constructed fusion genes encoding the HCV proteins core, NS2, NS3, NS4A, and NS5A conjugated to one half of YFP, and the NTFs IPOA5, IPO5, and XPO1 conjugated to the other half of YFP. Following transfection into 293T cells with fusion genes encoding pairs of potential viral protein-NTF interactors, cells were monitored for formation of fluorescent YFP. Multiple fusion genes were constructed to allow testing of all eight possible combinations of N- and C-terminal tagged pairs of potential interacting proteins (see S2 Figure). Fluorescence arising from representative combinations of the targeted HCV proteins and NTFs are shown in Fig. 2. The interactions of the remaining construct combinations are summarized in S1 Table. We observed the assembly of the fluorophore in cell culture when chimeric constructs containing core were expressed with IPOA5, IPO5, or XPO1 but not with a plasmid expressing the half YFP alone (Fig. 2E-H). This assay also detected interactions between NS2 and IPOA5 or XPO1 (Fig. 2I-L). NS3 showed interactions with IPOA5 and IPO5, but not with XPO1 (Fig. 2M-P, respectively), and NS5A appeared to specifically bind IPO5 (Fig. 2U-X). By contrast, NS4A, which based on our peptide array analysis does not bind NTFs, also failed to interact with NTFs using the BiFC assay (Fig. 2Q-T). It is important to note that none of the HCV fusion proteins or the NTF fusion proteins produced a fluorescent signal when produced in cells with the complementary half of the YFP molecule alone (Fig. 2A-C, 2H, 2L, 2P, 2T and 2X).

### Synthetic peptides bearing the putative nuclear transport signals are able to disrupt interactions between the HCV proteins and the NTFs

**Figure 3:**
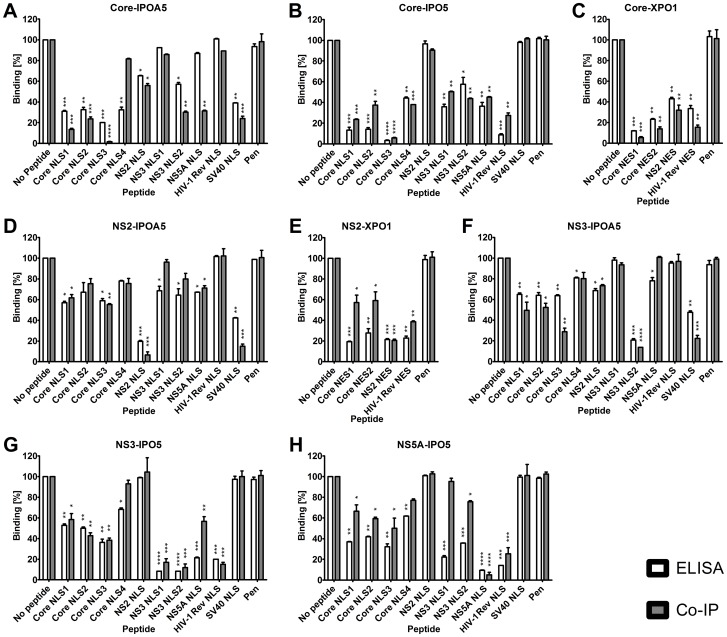
Analysis of HCV protein binding to NTFs in the presence of NLS and NES peptides. ELISA plates were coated with HCV proteins and the relative binding of the different NTFs was monitored by ELISA in the presence of 100 µM of the indicated peptide. For the Co-IP study, cells were transfected by plasmid encoding to V5 tagged HCV proteins and treated by the indicated peptides. After 48 h, cells were lysed and Co-IP using anti V5 antibody and blotted to enable detection of the different NTFs. Western blots were quantified using Multi Gauge v. 3.0. (A) Core-IPOA5, (B) Core-IPO5, (C) Core-XPO1, (D) NS2-IPOA5, (E) NS2-XPO1, (F) NS3-IPOA5, (G) NS3-IPO5, and (H) NS5A-IPO5. The y-axis represents the percentage of NTF bound to the HCV protein; the interaction without the peptide is set to 100%. Error bars represent ± standard deviation, n = 3. The significant difference between the untreated (no peptide) and peptide treated is indicated by: * p<0.05, ** p<0.01, *** p<0.001, **** p<0.0001.

Although the binding of HCV proteins to NTFs appears to be specific, some HCV proteins, such as core and NS5A, contain native unfolded domains that are promiscuous in their binding to host proteins [60], [61]. To examine the specificity of the interactions between HCV proteins and NTFs, we examined the ability of each putative NLS or NES peptide identified in Fig. 1 to disrupt the interaction between the HCV protein and the NTF, using an ELISA *in-vitro* and co-immunoprecipitation (Co-IP) in cell culture assays (Fig. 3, S3 and S4 Figure). The ELISA plates were coated with HCV proteins while NTFs and competitive peptides were added, and the fraction of bound NTF was measured using NTF specific antibodies. The Co-IP was conducted by expression of V5 tagged HCV proteins in the presence of competing peptides, followed by immunoprecipitation with V5 specific antibody and immunoblotting with specific NTF antibodies. Consistent with both peptide arrays (Fig. 1) and ELISA assays (S1 Figure), these studies revealed that all NLS peptides that bound IPOA5 were able to inhibit the interaction of core, NS2, or NS3 with IPOA5 (Figs. 3A, 3D, and 3F, respectively). Similarly, peptides that interact with IPO5 were able to inhibit the interaction of core, NS3, or NS5A with IPO5 (Figs. 3B, 3G, or 3H). Surprisingly, there was remarkable specificity of the interaction between importins and HCV proteins. We observed that the NLS peptide with the tightest binding to the NTF was not necessarily the best competitor interaction between the NTF and the HCV protein. Instead, the best peptide competitor for a given interaction between HCV protein and NTF was invariably derived from the corresponding HCV protein itself. For example, the NLS in NS2 was the best competitor for NS2-IPOA5 interaction even though its K_d(app)_ was five times higher than the K_d(app)_ of core NLS 3 for IPOA5. This “competition specificity” was not observed in interactions between HCV proteins and XPO1 *in-vitro* using ELISA; however, it was observed in cell culture in the Co-IP experiments (Figs. 3C and 3E). Using ELISA, the core NES 1 had the lowest K_d(app)_ for binding XPO1, and it was the best competitor for both the core- and NS2-XPO1 interactions. However, in the Co-IP assays in cell culture the competition was specific: the NS2 NES was the best competitor for the NS2-XPO1 interaction (Figs. 3C and 3E, respectively).

### HCV nuclear transport signals are functional in 293T cells

To determine whether NTF binding sites found in the HCV proteins were functional nuclear transport signals, we constructed reporter proteins each consisting of one of the HCV proteins NTF binding sites followed by two C-terminal, tandemly repeated GFP molecules. The double GFP moieties were required to keep the reporter from entering the nucleus, since a single GFP is small enough to diffuse freely through NPCs [34], [35]. As controls, constructs containing the reversed amino-acid residue sequence of the potential NLS (SLN) were also examined using the double GFP reporter assay (S5 Figure). These SLN reporters were predicted to not accumulate in the nucleoplasm. In addition, reporters to examine the functionality of the potential XPO1 binding domains were also constructed. These reporters consist of an SV40 NLS and a double GFP molecule followed by one of the various potential NESs at the C-terminus (S5 Figure). In the absence of a functional NES, this reporter will accumulate in the nucleus. However, a functional NES is predicted to export the reporter, leading to increased steady-state levels of the reporter in the cytoplasm.

**Figure 4:**
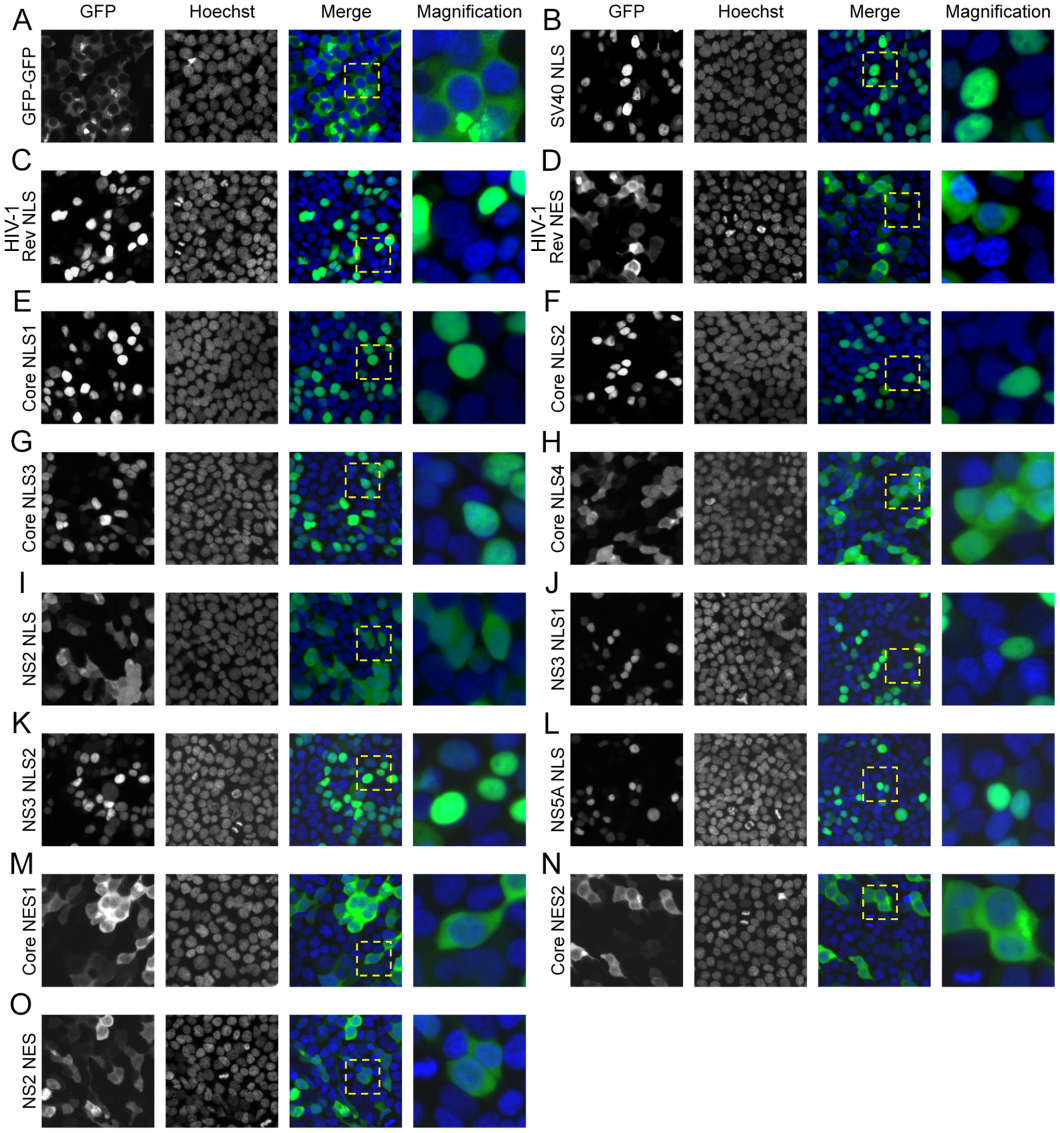
Functionality of the putative nuclear transport signals in cells. Cells were transfected by the vectors encoding a nuclear transport reporter consisting of a tandemly repeated double GFP fused to the indicated C-terminal nuclear transport signal (panels A-N). At 24 h following transfection, cells were fixed and stained with Hoechst to visualize the nucleus. Cells were examined using a Zeiss inverted Axiovert 200 M microscope to assess the localization of the GFP (green) and its relationship to the nucleus (blue). A hashed yellow square demarcates the magnified area shown in the right-most image of each panel. Fluorescent intensity was quantified using ImageJ v. 1.45 s and these data are presented in S6 Figure. Analysis of the reverse sequences of each transport signal as well as the mutant SV40 NLS was also performed and the results are shown in S7 Figure. The efficiency of the transport is summarized in Table 4.

The localization of these reporter constructs in cells was used to assess the functions of the various NTF binding domains (Fig. 4, S6 and S7 Figure). As shown in Fig. 4A, 293T cells producing the double GFP alone exhibited a cytoplasmic fluorescence signal and nuclear exclusion. Reporters containing the SV40 NLS or the HIV-1 Rev NLS, functional NLSs imported by IPOA5 and IPO5, accumulated in the nucleus (Figs. 4B and 4C, respectively). By contrast, a reporter containing a non-functional SV40 mutant NLS [62] remained in the cytoplasm and was excluded from the nucleus (S7A Figure). For the nuclear export positive control, we used an SV40 NLS, double GFP reporter containing a C-terminal HIV-1 Rev NES. As predicted, this reporter appeared primarily in the cytoplasm while its reverse NES (SEN) counterpart was concentrated in the nucleus (Fig. 4D and S7B Figure, respectively).

**Table 4:** Nuclear transport efficiency of double GFP cargo by the different nuclear transport signals.

Nuclear transport signal reporter	Localization	
	Nuclear	Cytoplasmic
GFP-GFP	−	+++
SV40 NLS	+++	−
HIV-1 Rev NLS	+++	−
HIV-1 Rev NES	+	++
Core NLS1	+++	−
Core NLS2	+++	−
Core NLS3	+++	−
Core NLS4	++	++
NS2 NLS	++	++
NS3 NLS1	+++	−
NS3 NLS2	+++	−
NS5A NLS	+++	−
Core NES1	+	++
Core NES2	+	++
NS2 NES	+	++
SV40 mut NLS	−	+++
Rev SEN	+++	−
Core SLN1	−	+++
Core SLN2	−	+++
Core SLN3	−	+++
Core SLN4	−	+++
NS2 SLN	−	+++
NS3 SLN1	−	+++
NS3 SLN2	−	+++
NS5A SLN	−	+++
Core SEN1	+++	−
Core SEN2	+++	−
NS2 SEN	+++	−

Quantified from the results presented in S6 and S7 Figures. The localization to the compartment was ranked as: +++ for strong, ++ for moderate, + for weak and − for no localization.

Upon examination of reporters containing the putative core NLSs or their reverse sequences (SLN), we observed that the core NLS 1-3 sequences robustly accumulated in the nucleus (Figs. 4E, 4F and 4G), while core NLS 4 (Fig. 4H) failed to concentrate the reporter solely in the nucleus and the reporter was visible in both the cytoplasm and the nucleus. When the SLN versions of these NLS sequences were used (S7C-F Figure) the reporter appeared entirely in the cytoplasm. The NS2 NLS, like core NLS 4, did not fully concentrate the reporter in the nucleus and it was present in both the nucleus and cytoplasm of the cell (Fig. 4I) while the NS2 SLN was localized only in the cytoplasm (S7G Figure). The two putative NLSs of NS3 and the NS5A NLS localized the reporter to the nucleus (Figs. 4J, 4K, and 4L, respectively) while the double GFP with the corresponding SLN sequences always remained in the cytoplasm (S7H-J Figure). Like the HIV-1 Rev NES control (Fig. 4D), the core NES 1, NES 2 and the NS2 NES (Figs. 4M, 4N, and 4O, respectively) were able to export the SV40 NLS double GFP out of the nucleus, whereas the corresponding SEN sequences were unable to mediate nuclear export (S7K-M Figure). Interestingly, the presence of functional nuclear transport signals within these membrane-associated HCV proteins would suggest a role for the nuclear transport machinery within the membranous web.

### HCV nuclear transport signals involved in multiple steps of HCV life cycle

**Figure 5:**
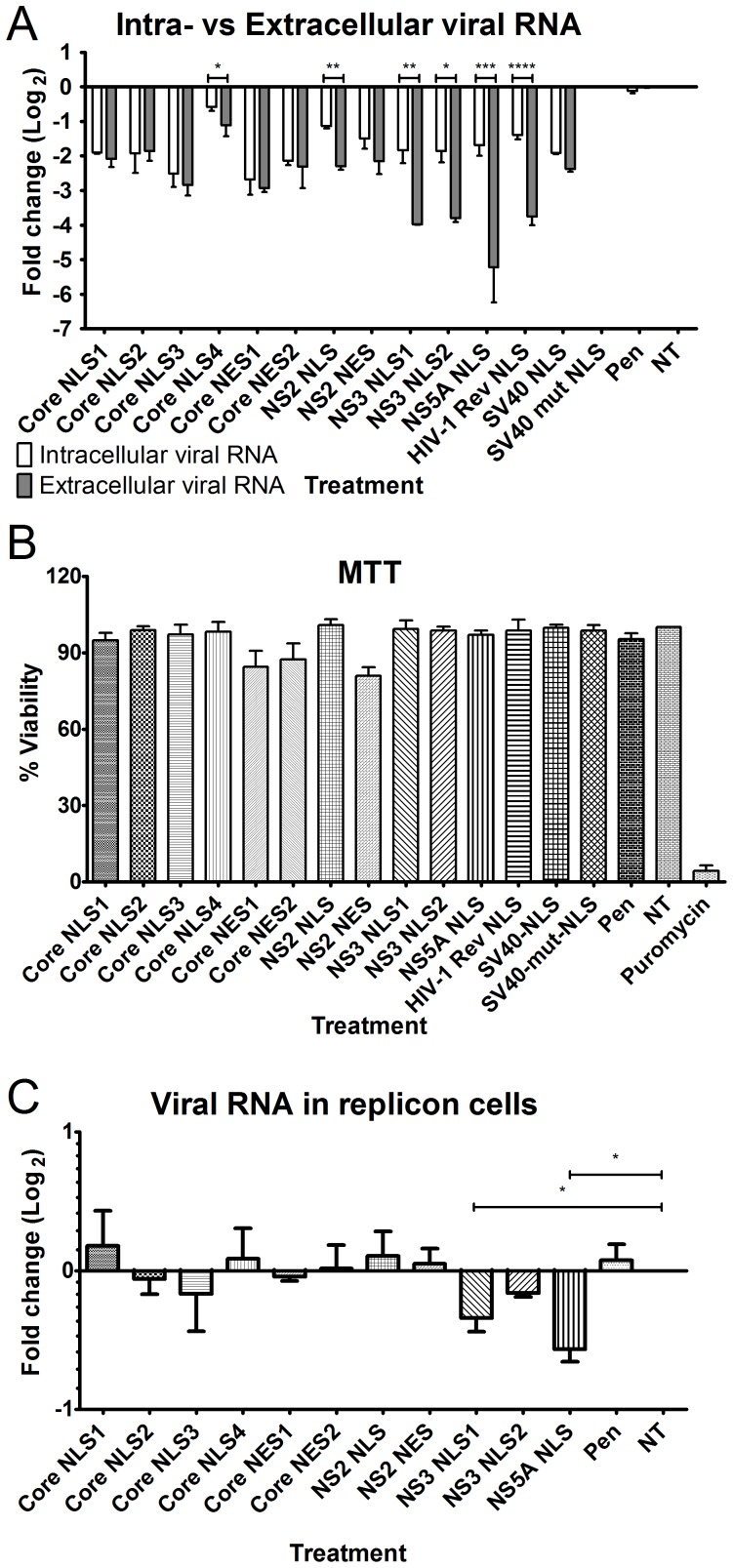
Effect of different nuclear transport signals bearing peptides on HCV infection. Cells were infected by HCV and treated with the indicated peptides. (A) Following 4 days of infection, virus titer was estimated by monitoring the levels of intracellular and extracellular viral RNA, using real time PCR. Significant differences between the intracellular and extracellular viral RNA levels are indicated by: * p<0.05, ** p<0.01, *** p<0.001, **** p<0.0001. (B) To assess the effect of the peptide treatment on cell viability, the treated and non-treated HCV infected cells were tested using an MTT assay. The viability was normalized to infected cells not treated with peptide. As positive control for toxicity in the MTT assay, Puromycin (Life Technologies A11138-02) was added to cells at a toxic concentration of 5 µg/ml. (C) Huh7.5-Neo/Luc-E.T. replicon cells were treated by the indicated peptides and the levels of viral RNA were monitored using real time PCR. The significant difference between untreated (NT) and peptide treated cells is indicated by: * p<0.05. Error bars represent ± standard deviation, n = 3.

The importance of nuclear transport on HCV replication was evaluated by examining the effects of peptides bearing the different nuclear transport signals on HCV infection in Huh7.5 cells by monitoring intracellular and extracellular viral RNA levels using real time qPCR (Fig. 5A). Our results indicate that the nuclear transport signals fell into two groups. The first group included core NLS 1–4, core NES 1 and NES 2, as well as NS2 NLS and NES. Treatment of cells with these peptides resulted in a similar fold reduction in the levels of intracellular and extracellular viral RNA, suggesting these peptides were likely inhibiting steps prior to or during viral replication rather than during virus assembly and egress (Fig. 5A). The second group included the NS3 NLS 1 and NLS 2, and NS5A NLS peptides. Treatment of cells with these peptides inhibited extracellular viral RNA production more profoundly than the level of intracellular viral RNA, and thus likely inhibited multiple steps in virus production including viral replication, assembly and egress (Fig. 5A). The observed inhibition by the Rev and SV-40 NLS peptide may be explained based on the results presented in Fig. 3 demonstrating that these peptides may interfere with multiple HCV proteins' interactions with NTFs. This result is most likely due to a partially overlap between their binding sites and the binding site of HCV proteins to the NTFs. None of the peptides, except the NES bearing peptide, showed any effect on the viability of the cells (Fig. 5B). The NES peptides showed a small reduction in the cell viability which may be due to the effect on host cell nuclear export [63].

We also examined the effect of the NLS peptides on viral RNA production in the Huh7.5-Neo/Luc-E.T replicon cell system. This replicon does not encode the core and NS2 proteins, but it does express NS3 and NS5A. Consistent with this structure of the replicon, we observed that treatment of Huh7.5-Neo/Luc-E.T replicon cells with the NS3 NLS1, NS3 NLS2, and the NS5A NLS peptides inhibited viral RNA production (Fig. 5C), while no significant changes in viral RNA levels were detected following treatment of cells with the core and NS2 NLSs. Importantly, these data support the specific inhibitory effects of the NLSs peptides. Of note, we observed that the effect of the NS3 and NS5A NLS peptides in the replicon system was not as large as detected in virally infected cells. It is unclear why this is the case, however, this effect may be due to the constitutive nature of viral RNA production in the replicon cells.

**Figure 6:**
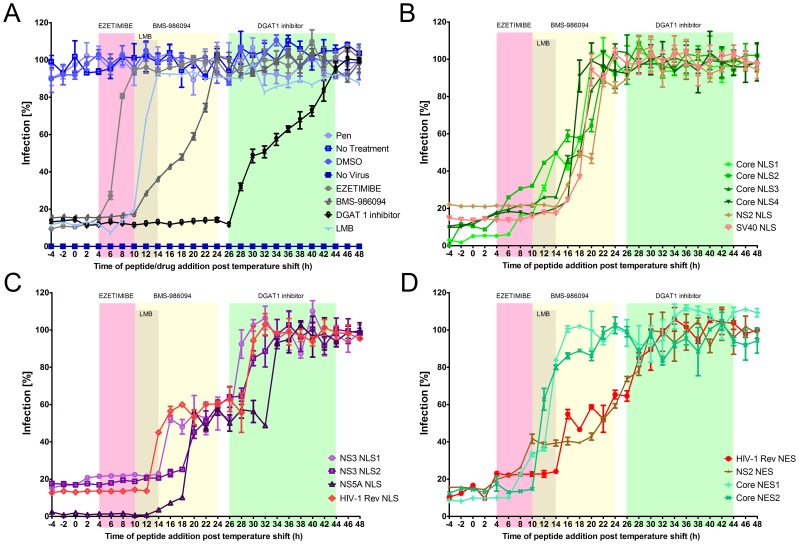
Nuclear transport signal-bearing peptides affect specific steps of infection: Time of addition experiments. In order to elucidate the time points in infection when the nuclear transport signals bearing peptides are functional, cells were synchronously infected and treated with the indicated peptides. Each time point (2 h intervals) represents a culture treated with the indicated reagent at the specified time post temperature shift (PTS) and initiation of HCV infection. All samples were fixed and probed with anti-NS5A antibodies to determine the percentage of infected cells relative to untreated cells at 72 h PTS. (A) Known inhibitors of HCV production (Ezetimibe (30 µM), LMB (18 nM), BMS-986094 (10 nM) and DGAT 1 inhibitor (10 nM)) and control (Pen peptide 100 µM, DMSO 2%, no treatment, and no infection) were examined. Groups of NLS and NES peptides appeared to yield similar inhibitory effects and are grouped in panels B-D (B) a group of NLS peptides (core NLS 1-4, NS2 NLS and SV40 NLS) inhibiting intracellular and extracellular viral RNA to approximately the same level, (C) the NLS peptides (NS3 NLS 1-2, NS5A NLS and HIV-1 Rev NLS) which further inhibited the extracellular virus and (D) the NES peptides (core NES 1-2, NS2 NES and HIV-1 Rev NES). Error bars represent ± standard deviation, n = 3.

Since the nuclear transport peptides seem to inhibit multiple steps of the HCV life cycle, we sought to define the timing of their actions by synchronizing viral infection and “bookmarking” the steps of infection using inhibitors that act at known points during the HCV life cycle. Huh7.5 cells were infected with JFH-1 HCV and maintained at 4°C for 4 hours (h) to synchronize the cells before shifting the temperature to 37°C. Post temperature shift (PTS), infected cells were incubated for various lengths of times prior to the addition of peptides or known inhibitors of HCV at 2 h intervals. The number of infected cells was monitored at 72 h PTS by immunofluorescence using anti-NS5A antibodies (Fig. 6). The number of cells infected at time 0 h was the same for all treatments and one infection cycle in the synchronized cells was completed by 48 h PTS. We know the cycle of viral infection in synchronized cells was approximately 48 h PTS since the ezetimibe curve did not show a biphasic response which would be expected if more than one cycle had occurred during the 48 h PTS (Fig. 6A). We deliberately selected the final time point of 72 h PTS to reflect the number of cells infected by a second round of infection. Peptides or known inhibitors added at the indicated time points during the first cycle of infection would have affected virus production. This would be reflected by the number of cells infected in the second round as measured at 72 h PTS. If the peptide or known inhibitor was added at a time point in the life cycle after it affects virus production, the virus production would have been uninterrupted and the second infection would have been observed as in untreated cells.

Ezetimibe is known to inhibit a late stage of HCV entry [64], and it lost its ability to inhibit HCV infection when added 4–10 h PTS, thus defining the last phase of entry (Fig. 6A). Leptomycin B (LMB) has been shown to inhibit HCV by inhibiting nuclear export mediated by XPO1, and this inhibitory effect was lost at 8 h post infection [39]. In our study, the inhibitory effect of LMB was lost 10–14 hours PTS (Fig. 6A). BMS-986094, an inhibitor of NS5B polymerase, lost its ability to inhibit HCV infection when added 10–24 h PTS, thus defining the period of viral RNA synthesis (Fig. 6A). The diglyceride acyltransferase 1 (DGAT1) inhibitor is known to inhibit the late assembly phase of the viral life cycle [65]. This inhibitor lost its ability to inhibit HCV when added 24–44 h PTS (Fig. 6A), thus defining the phase of viral assembly. The controls of Pen (Penetratin, an inactive peptide), DMSO and no treatment had no effect on infection (Fig. 6A). The results broadly define the various stages of the HCV life cycle and they were used as “bookmarks” to further determine the points at which the peptides that inhibit nuclear transport signals had their greatest effect on the HCV life cycle.

Interestingly, the majority of the peptides produced biphasic inhibition curves indicating that they act at multiple points during the HCV life cycle. The core NLS 1 and NLS 2 peptides began to lose inhibitory activity during the early stage of infection (4–10 h PTS) (Fig. 6B). This time period is immediately after the inhibitory period defined by ezetamibe, which inhibits viral entry. This result is very intriguing since both core and NS2 are known to function during late steps of the viral life cycle. However, this assay does not necessarily suggest a direct effect of these proteins on early steps in the viral life cycle, such as entry or replication; rather, these proteins may interact with NTFs early in infection, which may affect the later functions of these proteins. The core NES 1 and NES 2 peptides began to lose their ability to inhibit HCV infection immediately after the first 2 NLSs of the core lost some of their effect (∼6–10 h PTS) (Fig. 6D). These results imply that there is an early stage in HCV infection that involves the NLS and NES sequences of the HCV core. This correlates with the results of Cerutti, A et al. [39] and our results (Fig. 6A) showing that LMB inhibits HCV infection only if added early during infection.

All of the core NLS peptides, as well as the NS2 NLS peptide appear to exert the majority of their ability to inhibit HCV infection during the replication phase of infection (12–22 h PTS) (Fig. 6B), as inferred by the comparison to the inhibitory profile produced by BMS-986094, a known inhibitor of the NS5B (Fig. 6A). These results imply that NLS sequences within HCV proteins are important during this stage of the viral life cycle. However, it remains to be determined whether these peptides are affecting replication or their actions are merely coincident with replication. For example, given our previous results showing NPC proteins within the membranous web [32], one possibility is that the peptides inhibit the transport of these HCV proteins into the membranous web rather than directly affecting the replication complex.

The most impressive biphasic inhibition curves were produced by NS3 NLS 1, NS3 NLS 2, and NS5A NLS (Fig. 6C). These peptides exert about 40–50% of their ability to inhibit HCV during the replication phase (12–20 h PTS) and the other 50–60% during the assembly phase (26–34 h PTS). The first step of inhibition observed during replication is similar to the inhibition obtained in the replicon cells with the NS3 NLS1 and NS5A NLS peptides (Fig. 5C). This correlates with our results showing that these peptides inhibit HCV RNA in the supernatant of cells to a greater extent than they inhibit HCV RNA within the cells (Fig. 5A). It is important to note that these results do not necessarily suggest a direct effect on replication or assembly, but on functions during this time that affect the life cycle of the virus. Additionally, this biphasic effect is not due to a second cycle of infection since the early inhibitors ezetimibe and LMB do not show a similar biphasic effect, suggesting this is still the first cycle of the infection.

Examining the effect of the NES peptides we also see two types of response. The first, as mentioned above, concerns the core NES 1 and NES 2, which suddenly lose their ability to inhibit HCV early in the viral life cycle (10–14 h PTS) (Fig. 6D). The second response is shown by the NS2 NES and the control HIV-1 Rev NES which exhibit their ability to inhibit HCV in a biphasic curve, first early in the viral life cycle (8–16 h PTS) and then gradually lose their ability to inhibit HCV infection between 18–30 h PTS (Fig. 6D).

These results, taken in consideration with our previous published results [32], lead us to conclude that the nuclear transport machinery is intimately involved in multiple steps of the HCV life cycle.

### Relocalization of nuclear transport signal-containing cargos in HCV infected cells

**Figure 7:**
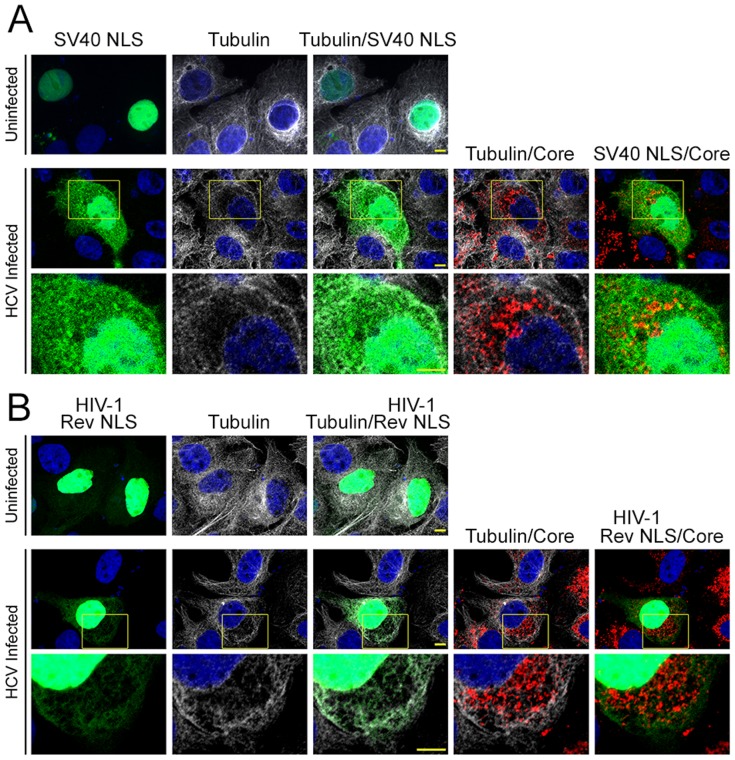
Transport of NLS-containing cargos into the membranous web is NTF-specific. Uninfected cells and cells infected with HCV were transfected with a plasmid encoding a transport reporter consisting of a tandemly repeated GFP tagged with a C-terminal SV40 NLS (A) or HIV-1 Rev NLS (B). At 24 h post transfection, cells were probed with anti-HCV core antibodies to monitor infection and with Hoechst to detect nuclei. Tubulin staining was used as a marker for the membranous web since it was excluded from it.

We recently reported that during HCV infection, Nups are recruited to the replication sites of HCV, namely the membranous web [32]. We suggested that this recruitment results in the creation of selective transport to and from the membranous web. Since SV40 NLS and HIV -1 Rev NLS are signals of transport cargos by IPOA5 and IPO5, respectively, we used these NLSs to construct NLS-double GFP reporters (see Fig. 4, S5-S7 Figures) and monitor their localization in the HCV infected cells (Figs. 7A and 7B, and S8 Figure). As can be seen in Figs. 7A and 7B, both the SV40 NLS-GFP and the HIV-1 Rev NLS-GFP reporters were localized exclusively in the nuclei of uninfected cells. In similar transfections of HCV infected cells, some of the SV40 NLS-GFP reporter (an IPOA5 cargo) was additionally localized in the cytoplasm, including regions enriched for HCV core and lacking tubulin staining, feature characteristics of the membranous web [32]. Interestingly, in contrast, when infected cells were transfected with the HIV-1 Rev NLS GFP reporter (an IPO5 cargo), the reporter was visible in both the cytoplasm and the nucleus but was excluded from the regions of the membranous web (Fig. 7B). These results suggest HCV NLS sequences play distinct roles during the HCV life cycle.

We therefore investigated, using the dual GFP reporter systems, whether the nuclear transport signals of the HCV proteins could also mediate transport into the membranous web. As predicted, GFP reporters containing NS3 NLS 1 and NS5A NLS (IPO5 cargos) were imported into nuclei of uninfected cells. However, in HCV infected cells, both of these reporter proteins also showed some diffuse cytoplasmic signals and were excluded from regions containing HCV core (S8A and S8B Figures). This pattern is similar to the localization pattern seen with HIV-1 Rev NLS reporter (Fig. 7B), another IPO5 cargo. The rest of the HCV NLSs reporters showed a relatively even distribution of the GFP signal in the cytoplasm (see example core NLS1 S8C Figure). All of the HCV derived NLS-GFP reporters were found in the nuclei of both uninfected and HCV infected cells, indicating that HCV infection did not significantly interfere with nuclear transport. When the nuclear export signals, NS2 NES and core NES 1 and NES 2 reporters, were tested, no significant changes in the localization of GFP were observed between the non-infected and the HCV infected cells (data not shown).

## Discussion

Positive strand RNA viruses generally replicate and assemble in the cytoplasm of infected cells, however many virally encoded proteins have been shown to contain nuclear transport signals and to interact with the nuclear transport machinery. The prevalence of nuclear transport signals in flaviviruses led us to investigate the role of the nuclear transport machinery using HCV as the prototype. Previously, we have shown that NPC proteins are recruited to the membranous web, interact with HCV proteins, and are required during HCV infection [32]. We have proposed that the presence of the NPC proteins in the membranous web contributes to its membrane architecture and facilitates the transport of viral and host proteins from the cytosol and into viral replication and/or assembly compartments. In this paper, we have examined the transport component of this model.

Similar to other positive-strand RNA viruses, HCV was previously reported to contain several NLS sequences in core and NS5A [41], [50]. In addition, NS3 was shown to bind IPOA5 in the yeast two hybrid system [66]; however, no NLS was characterized. NES sequences were also identified in the core protein [39], suggesting that this protein may be able to shuttle between the cytoplasm and the nucleus. Moreover, HCV has been shown to interact with several NTFs and Nups [32], [52]. These observations were perplexing, as the HCV life cycle does not have a clear nuclear step. In this study, we discovered several previously unidentified nuclear transport signals in HCV proteins and performed a series of experiments to evaluate the functionality of the newly and previously identified NLSs and NESs from HCV proteins. Moreover, we showed that these nuclear transport signals are required for viral infection and that specific nuclear transport signals are functional at different stages in the HCV life cycle. Finally, in support of our previously proposed model, we showed that specific NPC-mediated transport pathways are active in transporting proteins into the HCV-induced membranous web (Fig. 7 and S8 Figure).

We examined the involvement of the nuclear transport machinery during the HCV life cycle in detail. To identify regions in HCV proteins that contain potential NLS or NES domains, we probed a peptide array representative of the HCV polyprotein to identify NTF binding peptides. This analysis confirmed known nuclear transport signals in core and NS5A, as well as importin and exportin binding peptides in the NS2 and NS3 proteins. Importantly, we confirmed, using GFP reporter proteins, that each of the identified NTF-binding peptides was capable of functioning as a nuclear import or export signal, albeit core NLS4 and the NS2 NLS only weakly supported nuclear import. These results support their presumed functional interaction with the nuclear transport machinery.

We examined the affinity of each nuclear transport signal for their cognate NTF. Interestingly, some of the nuclear transport signals were promiscuous in their ability to bind NTFs while others were specific for transport factor binding. The core NLS1-3 and the NS3 NLS2 bound both IPOA5 and IOP5. Core NLS4 and the NS5A NLS also bound both NTFs but showed a significantly higher affinity (5- and 200-fold, respectively) for IPO5. By contrast, the NS3 NLS1 bound only IPO5 and the NS2 NLS bound specifically to IPOA5. These results suggest that viral proteins can potentially utilize several nuclear import pathways. A similar use of multiple nuclear import pathways has been shown previously in HIV-1 where both importin and transportin pathways are required to transport the pre-integration complex into the nucleus [67]. For all of the potential NLSs, the reverse sequence did not show binding to the NTFs, indicating that the binding is specific for the sequence and not due to the charge of the domain. Consistent with these results, various *in vitro* and in cell culture assays, including ELISA, BiFC, and Co-IP, detected the core and NS3 proteins bound to both IPOA5 and IPO5, while NS5A only bound to IPO5 and NS2 only to IPOA5 (S1 and S4 Figures, Figs. 2 and 3).

Our ELISA and Co-IP assays allowed us to assess the contributions of various NLS sequences to the interactions of HCV proteins with the NTFs. We observed that control NLS peptides that specifically bind IPO5 (Rev-NLS) or IPOA5 (SV40 NLS), efficiently competed with HCV proteins for binding to these NTFs. For example, the core-IPO5 interaction was reduced by the Rev-NLS but not the SV40 NLS, while the core-IPOA5 interaction was inhibited by the SV40 NLS and not the Rev-NLS. Likewise, HCV NLSs that only bind IPO5 or IPOA5 (NS3-NLS1 and NS2-NLS, respectively) demonstrated similar specificities, and only inhibited the interactions of HCV proteins with their cognate NTFs. By contrast, those HCV NLSs that interact with both IPO5 and IPOA5, such as core NLS1-3, broadly inhibited the interactions of the NLS-containing HCV proteins with both NTFs. These latter results suggest that the various HCV NLSs share binding sites on the NTFs. Surprisingly, the efficiency of a given NLS peptide to inhibit the interactions of an HCV protein with a specific NTF was not a direct function of its binding affinity for the NTF. For example, the core NLS3 (K_d_∼17.5 nM) binds more avidly to IPOA5 than the NS2 NLS (K_d_∼79.3 nM), yet the NS2 NLS was a more potent inhibitor of the interaction between the NS2 and IPOA5 (see Table 3 and Fig. 3). In general, we observed the strongest competition between a specific HCV protein and an NTF when the competing peptide was derived from the same HCV protein. These results suggest that the HCV NLSs bind to distinct, but overlapping, regions of the NTFs.

The ability of cell-penetrating NLS peptides to inhibit the binding of HCV proteins to NTFs allowed us to investigate the role of these interactions in viral infection. When examining the effect of the NLS peptides on the intracellular and extracellular viral RNA, we observed that each of the peptides tested inhibited viral RNA production and produced one of two phenotypes. Treatment of cells with one group of peptides led to similar decreases in both intracellular and extracellular viral RNA levels. This group included the core NLSs (NLS1-4), the NS2 NLS, and the IPOA5-specific SV40 NLS. The coincident decreases in intracellular and extracellular viral RNA are most consistent with these NLSs disrupting HCV replication, or steps prior to or during replication. A second group of peptides also inhibited intracellular viral RNA levels, but had significantly greater effect on extracellular viral RNA levels. This group included the NS3 and NS5A NLSs as well as the IPOA5-specific HIV-1 Rev NLS. Interestingly, these NLSs specifically bind to IPO5. These results lead us to conclude that IPO5 transported proteins play a role during both the replication and assembly/release of the virus.

Our hypothesis that IPOA5 and IPO5 may contribute to distinct processes during the HCV life cycle was further supported by the results of experiments examining the effects of the inhibitory NLS/NES peptides on the HCV life cycle in synchronously infected cells (Fig. 6). Treatment of cells with peptides capable of binding IPOA5 (core NLS1-4, NS2 NLS, or SV40 NLS) at various times following infection revealed that each of these peptides inhibited infection if added prior to 20 h PTS (Fig. 6B). Importantly, this profile of inhibition is similar to that seen following treatment of infected cells with NS5B RNA polymerase inhibitor BMS-986094 (Fig. 6A). We conclude that these peptides were inhibiting during the time of viral RNA replication through their ability to dissociate specific HCV protein-NTF interactions.

A second group of peptides that bind to IPO5 (NS3 NLS1, NS3 NLS2, NS5 NLS, and HIV-1 Rev NLS) exhibited a more complex pattern of inhibition (Fig. 6C). In these cases, the inhibitory effects of the peptides were only partially lost when added at 12–20 h PTS and maintained at the same level up to 26 h PTS. This is similar to the effect seen in the replicon cells (Fig. 5C) suggesting an inhibitory effect during the replication stage. The second loss of the inhibitory effect of these peptides was in the 28–36 h PTS time frame, which is correlated with viral assembly as delineated using similar assays with the assembly inhibitor DGAT-1 (Fig. 6A). Thus, as we detected by monitoring viral RNA levels (Figs. 5A and 5C), IPO5-HCV protein interactions appear to be required during both viral RNA replication and a post replication assembly step. Furthermore, the presence of IPO5 specific NLSs in NS3 and NS5A are consistent with their proposed roles of these proteins during both viral RNA replication and viral assembly [68], [69].

The NES peptides also showed inhibitory effects on viral production in synchronously infected cells (Fig. 6D). Interestingly, both of the phenotypes observed with the NLS peptides were also detected with the NES peptides, with the specific effects being dependent on the source of the NES. The core NES 1 and 2 showed similar inhibitory profiles as seen with LMB (an inhibitor of XPO1), which again is consistent with a specific block of viral RNA replication (Fig. 5A). The NES 2 of core protein is likely not exposed in the context of virus since this domain is in the membrane [70]. The activity seen with the core NES 2 peptide could be attributed to overlapping of its binding domain with the binding domain of the core NES 1 on the XPO1. We observed small effects exerted by two out of the four core NLS domains (NLS 1 and NLS 2) at 4–10 h PTS, prior to the timing of the effect of the core NESs at 10–16 h PTS (Figs. 6B and 6D). This supports the results of a previous study, which provided evidence that core enters the nucleus early in infection [39]. The NS2-NES and the HIV-1 Rev NES appear to block events required for both viral RNA replication and post replication early assembly steps, similar to the IPO5 NLSs. These results suggest that, while these peptides can each bind the same NTF (XPO1) and function in nuclear export, their inhibitory effects are likely dependent on their ability to disrupt specific cargo-NTF interactions.

**Figure 8:**
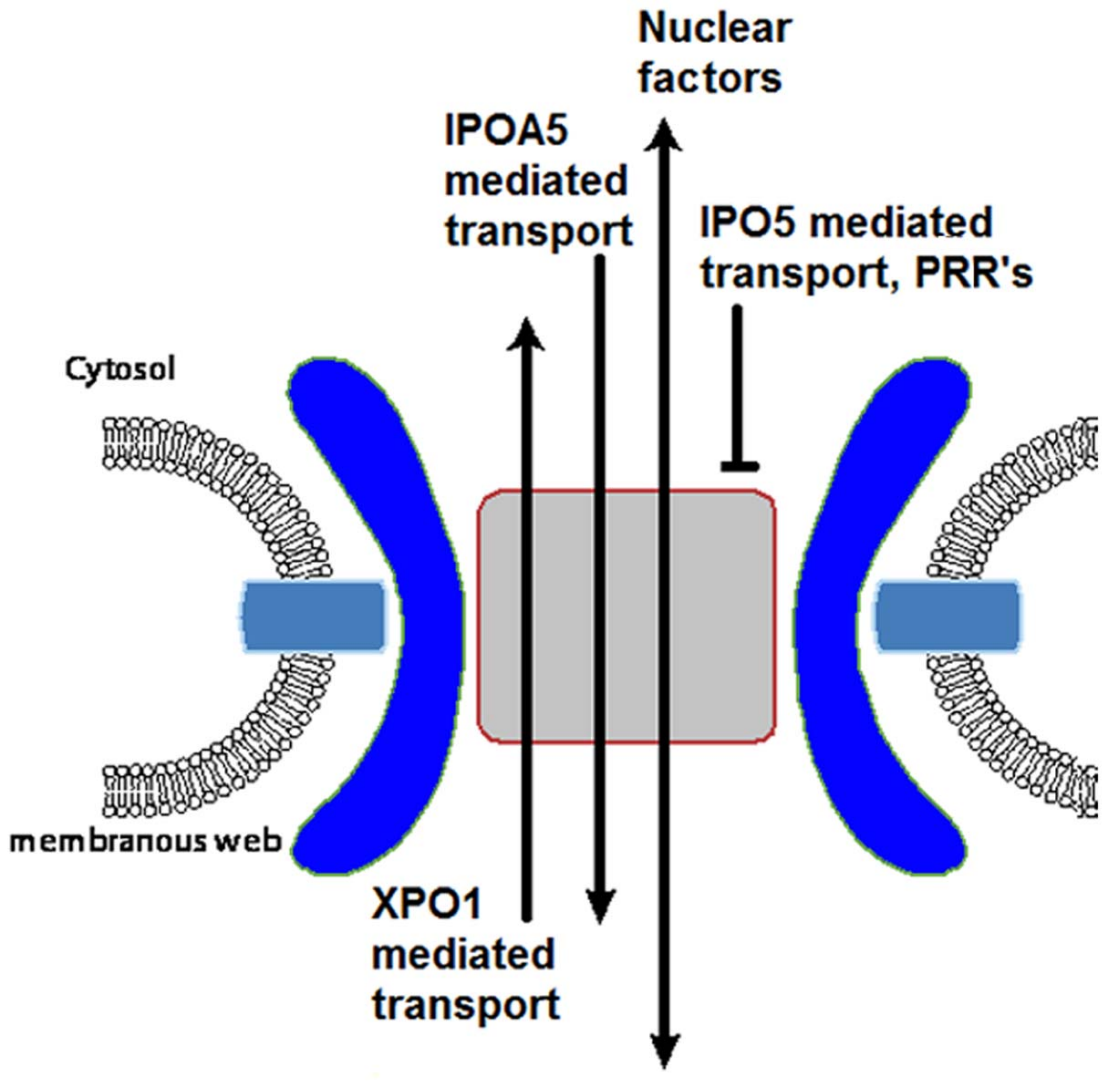
Model for the function of membranous web transport in HCV infection. NPC-like structures positioned in the membranous web of HCV-infected cells are proposed to form channels across double membrane structures of the membranous web. These NPC-like structures would enable selective transport of proteins containing NLS and NES sequences, such as HCV core, NS2, NS3, NS5A and host nuclear proteins from the surrounding cytosol across double membrane structures of the membranous web. The NPCs associated with the membranous web NPCs are also predicted to discriminate between NTFs, selectively importing IPOA5 cargo while excluding IPO5 cargos and proteins lacking NLS sequences, such as PRRs, from HCV replication and assembly sites.

Our data suggest that nuclear transport signals in several HCV proteins functionally interact with NTFs and that they are required for viral replication. However, with the possible exception of core [39], we do not envisage that HCV proteins use these interactions with the nuclear transport machinery to facilitate nuclear import. Instead, we propose that HCV protein-NTF interactions function in the context of the membranous web through their interactions with NPC proteins. We have previously shown that following HCV infection, Nups are recruited to the membranous web [32]. Here, we proposed that Nups may function in the formation of membrane microdomains and in forming web-associated NPCs, which would regulate transport across the double membranes that separate viral replication centers form the surrounding cytoplasm. Direct support for our hypothesis was provided by experiments showing that the SV40-NLS-GFP reporter (an IPOA5 cargo) has access to the interior of the membranous web (Fig. 7; see also [32]). HCV NLSs that interact with IPOA5 also can facilitate transport of reporters to the membranous web (S8 Figure; and data not shown). Thus, one function of the HCV NLSs could be to direct these HCV proteins to the membranous web were they can contribute to viral replication (Fig. 8). Furthermore, the interactions of the NTFs with the HCV proteins prior to entering the membranous web could function to restrict interactions of the HCV protein until these proteins reach replication and assembly centers (Fig. 8). Such a mechanism for NTFs has been proposed for capsid assembly in papovaviruses [71]. Since the HCV proteins containing NLSs are integral or peripherally-associated membrane proteins, we predict that these proteins use the NTFs and NPCs to access the interior of the membranous web by a mechanism similar to membrane proteins moving from the endoplasmic reticulum/outer nuclear membrane through the nuclear pore membrane to the inner nuclear membrane [72]. The ability of HCV proteins to recruit the nuclear transport machinery for the purpose of facilitating assembly of viral replication in the membranous web seems more likely than a role for these interactions in modulating nuclear transport, as we were unable to observe any significant changes in nuclear import in the infected cells (Fig. 7 and S8 Figure).

Strikingly, in contrast to IPOA5 reporters, transport reporters containing an IPO5-specific NLS, such as Rev-NLS-GFP and NS3 NLS1-GFP (Fig. 7 and S8A Figure), were largely excluded from the membranous web. These IPO5-specific NLS reporters were, however, capable of entering the nucleus. We interpret these results to suggest that the membranous web NPCs may have transport properties distinct from those of the nuclear envelope. Consistent with this idea, it has been proposed that structural and compositional changes in NPCs can alter the specific transport properties of NPCs [73], [74]. Notably, such changes can specifically inhibit transport mediated by the yeast counterpart of IPO5 while not affecting the IPOA5 counterpart (Fig. 8 and [74]). If IPO5 import into the membranous web is restricted, what function might the IPO5-specific NLSs play in the HCV life cycle? It is possible that the interactions of IPO5 with the HCV proteins play a non-transport role. NTFs have been proposed to function as molecular chaperones [75] and to contribute to the structure and assembly of the NPC [76], [77]. Similarly, NTFs could directly contribute to the structure of viral replication centers or virion assembly. Alternatively, interactions of HCV proteins with IPO5 could serve to negatively regulate the access of certain HCV proteins to regions of the membranous web and thus contribute to the fidelity of virus production.

## Materials and Methods

### Cell cultures

Monolayer adherent HEK293T and Huh7.5 cells were grown in Dulbecco's modified Eagle's medium (DMEM) (Sigma) supplemented with 10% (v/v) fetal bovine serum (FBS) (Sigma) and 1% penicillin streptomycin (PS) (Sigma). Huh7.5 media was also supplemented with 1% MEM non-essential amino acids (MEM-NEAA) (Gibco). Cells were incubated at 37°C in a 5% CO_2_. Huh7.5-Neo/Luc-E.T replicon cells were grown in the same media as the Huh7.5 cells with the addition of 250 µg/ml G418. The HEK293T were from ATCC. The Huh7.5 cells were a generous gift from Charles M. Rice (The Rockefeller University, New York, New York, USA) [78]. The replicon system was a generous gift from Ralf Bartenschlager (University Hospital Heidelberg, Heidelberg, Germany) [79].

### Infection

JFH-1 virus was gift from Takaji Wakita (National Institute of Infectious Diseases, Tokyo, Japan).

- Infection of Huh7.5 cells with JFH-1 for non-synchronized cell studies.
  Huh7.5 cells were seeded at a density of 4×10^5^ cells/well in a 6-well plate. The cells were washed with 1X PBS 24 h post plating, infected with 3 genomic equivalents of serially passaged JFH-1 and incubated at 37°C in a 5% CO_2_ for 4 h. Following the incubation, the cells were washed 3 times with DMEM containing 1% PS and incubated in DMEM containing 10% FBS, 1% PS, and 1% MEM-NEAA until harvested for the specific assay.
- Infection protocol for synchronized cell cultures used in the time of addition studies.
  For the time of addition experiments, cells were seeded at a density of 6.25×10^4^ cells/well in a 96-well plate and infected with 0.1 genomic equivalents of serially passaged JFH-1 virus on ice: cells then were incubated at 4°C for 4 h. Following this incubation the cells were washed three times in DMEM 1% PS and incubated with DMEM 10% FBS 1% PS 1% MEM-NEAA at 37°C in a 5% CO_2_ until fixed, and stained for NS5A and DAPI at 72 h PTS to calculate the percentage of infected cells.

### Peptide arrays

A consensus sequence of HCV Genotype 1 polyprotein was used to design consecutive fifteen amino-acid residue peptides with eight residue overlaps along the complete polyprotein sequence. Peptides were synthesised by Intavis Bioanalytical Instruments (Koeln, Germany) using a CelluSpots peptide array synthesis technology. Peptide arrays were incubated in blocking buffer (TBS containing 0.05% tween 20 (v/v) and 10% skim milk (g/v)) overnight at 4°C on a gentle shaker. Following the blocking, the peptide arrays were washed three times with washing buffer (TBS containing 0.05% tween 20 (v/v)) for 5 min at room temperature on a gentle shaker. The peptide arrays were then incubated for 4 h with cell lysate from two T-75 flasks of fully confluent cells lysed by lysis buffer (10 mM Tris-HCl, pH 7.5, 150 mM NaCl, 2 mM EDTA, 0.1% Triton-X100, 1 mM PMSF, 2 µg/ml aprotinin, 2 µg/ml leupeptin and 0.1 units/µl RNasin) mixed with blocking buffer (ratio 1∶1 v/v) and incubated at room temperature on a gentle shaker. Following this incubation, peptide arrays were rinsed with washing buffer for 30 sec and then washed five times in washing buffer and each wash lasted for 5 min at room temperature on a gentle shaker. Following washing, the peptide arrays were incubated with the primary antibody as indicated in Fig. 1 (1∶100 dilution) for 4 h at room temperature on a gentle shaker. Arrays were then rinsed, washed as described above, and incubated with a complementary HRP conjugated secondary antibody (1∶1000) for 2 h at room temperature on a gentle shaker. The peptide arrays were then rinsed and washed as described above followed by initiation of HRP chemiluminescence with ECL detection reagent (GE Healthcare, RPN2106) and the signal was detected using Fuji RX film (Fujifilm, 47410 08399).

### Peptide synthesis

Synthetic peptides were manufactured by GL Biochem Ltd (Shanghai, China). The sequences are shown in Table 2. Peptides were synthesized conjugated to a pentratin sequence (Pen) [55] to ensure cell permeability. The HIV-1 Rev NLS was not conjugated to penetratin since it is known to be cell permeable [80]. All peptides were purified by the manufacturer to >85% purity using HPLC.

### Cloning of constructs

All HCV genes used in this study were cloned into several vectors. For detailed information see S1 Methods.

### Expression and purification

All HCV and NTF genes were cloned into E. coli expression vectors and expressed and purified as detailed in S1 Methods.

### ELISA-based binding assays and competition

Protein-peptide and protein-protein binding were estimated using an ELISA-based binding assay as described previously [81]. Briefly, ELISA plates were coated with 200 µl of a 10 µg/ml of synthetic peptide or recombinant protein in a carbonate buffer (0.05 M Na_2_CO_3_/0.05 M NaHCO_3_, pH 9.6) overnight at 4°C. After incubation, the solution was removed, and the plates were washed three times with PBS. Plates were blocked with 200 µl of 10% BSA (Sigma) in PBS (w/v) for 2 h at room temperature. After rewashing with PBS, the indicated protein was added and incubated for an additional 2 h at room temperature. Following three washes with PBS, the primary antibodies—mouse anti-HIS antibody (1∶2000) to test NTF binding to peptides, and specific anti-NTFs antibodies (1∶200) to test protein-protein binding—were added for 2 h at room temperature. The concentration of bound molecules was estimated after the complementary secondary HRP conjugate antibody (1∶5000) was added for 1 h at room temperature. The amount of bound HRP was estimated by addition of TMB substrate and monitoring of the product's optical density (OD) at 450 nm using an ELISA plate reader. Each measurement was performed in duplicate. For competition assays, the indicated peptides were added at different protein to peptide ratios together with the NTFs.

### BiFC

HEK293T cells were seeded on coverslips and transfected with different combinations of the described BiFC vectors using lipofectamine 2000 reagent (Invitrogen, 11668019). Each interaction was performed for all eight vector pair combinations, encoding each of two proteins tested for their interaction. At 36 h post transfection, cells were fixed with 3.76% formaldehyde in PBS at room temperature (Sigma, F8775-500ML) for 10 min then permeablized in 0.2% Triton X-100 (VWR, CA97062-208) for 2 minutes. Cells were then washed three times with 1X PBS, stained with Hoechst (1∶5000) in 1X PBS for 5 min, and then washed three times with 1X PBS. Cells were then examined using a Zeiss inverted Axiovert 200 M microscope and 40x/0.75 objective lens to detect Hoechst and YFP. At least ten random fields of cells were imaged for each combination. Channels were merged using ImageJ software (National Institutes of Health). Photoshop Elements 10 (Adobe) software was used to adjust brightness and contrast levels for individual images and assemble images into figures.

### Co-Immunoprecipitation

HEK293T cells were transfected with the different HCV protein expression constructs described above using lipofectamine 2000 reagent (Invitrogen, 11668019). At 48 h post transfection, cells were lysed with ice cold lysis buffer (10 mM Tris-HCl, pH 7.5, 150 mM NaCl, 2 mM EDTA, 0.1% Triton-X100, 1 mM PMSF, 2 µg/ml aprotinin, 2 µg/ml leupeptin and 0.1 units/µl RNasin) and incubated on ice for 15 min. Lysates were clarified by centrifugation at 18,800 g at 4°C for 15 min. The resulting supernatant was incubated with anti-V5 antibody for 4 h at 4°C. Protein G Sepharose beads were then added and incubated overnight with mixing. Beads were washed eight times with wash buffer (50 mM Tris-HCl, pH 7.5, 150 mM NaCl, 0.05% Triton-X100). After each wash, the beads were centrifuged at 2,400 g for 5 min at 4°C and the supernatant was removed. After the last wash, the entire supernatant was removed and beads were stored at −20°C. When peptides were used to disrupt HCV proteins interactions with nuclear transport factors, 125 µM of the indicated peptide was added to the culture 12 h post transfection. Protein samples were run on SDS PAGE and transferred to nitrocellulose membrane. Membranes were blocked with TBS-T (TBS containing 0.1% tween 20) containing 5% skim milk overnight at 4°C, followed by incubation with the indicated primary antibodies (1∶200) in blocking solution at room temperature for 4 h. Following incubation with primary antibodies, the membranes were washed five times with TBS-T (0.1% tween 20) for 15 min each and then incubated for 2 h with HRP-conjugated secondary antibodies. Membranes were washed five times with TBS-T for 15 min each followed by initiation of HRP chemiluminescence with ECL detection reagent (GE Healthcare, RPN2106). Signals were detected using Fuji RX film (Fujifilm, 47410 08399).

### Localization of nuclear transport signals-double GFP reporter

#### In HEK293T cells

Nuclear transport signals-double GFP constructs were transfected into HEK293T cells using lipofectamine 2000. At 24 h post transfection cells were fixed, stained, and visualized for localization of the GFP. For detailed information see S1 Methods.

#### In Huh7.5 cells

Localization studies in Huh7.5 cells were performed as described above with the following modification; the constructs were transfected into infected or uninfected Huh7.5 cells using lipofectamine 2000 in a reverse transfection. For detailed information see S1 Methods.

### RNA isolation and reverse transcription

#### Intracellular

RNA was extracted from cells using Trizol (Invitrogen, 15596-018) according to the manufacturer's specifications and cDNA was synthesized using random primers (Invitrogen, 48190-011) and MMLV reverse transcriptase (Invitrogen, 28025-013) as described by the manufacturer.

#### Extracellular

For the analysis of HCV RNA levels released from cells, RNA was isolated from 200 µl of culture supernatant of infected cells using a High Pure Viral Nucleic Acid Kit (Roche, 11858874001) according to the manufacturer's specifications. The cDNA was synthesized using superscript III (Invitrogen, 18080-044) and an HCV specific primer (see HCV reverse primer sequence S2F Table) also according to the manufacturer's specifications.

### Quantitative real-time PCR (qPCR)

Quantitative PCR was performed using TaqMan Master Mix with HCV primers and labeled probe (S2F Table) on an ABI 7900 real time PCR machine. Primers and probe for qPCR were designed using primer3 software (S2F Table). PCR efficiency for each primer was determined using the slope of a standard curve derived from qPCR analysis of cDNA serial dilutions. To obtain the relative abundance of specific RNAs from each sample, values were corrected for the specific PCR efficiency of the primer, and normalized to HPRT transcript levels. The HPRT levels were determined using huHPRT VIC-MGB primer probe mix (Applied Biosystems 4326321E).

### Time of addition studies

The time of addition experiments were done based on similar experiments investigating the life cycle of HIV-1 [81] and HCV [64]. Briefly, Huh7.5 cells were infected as described above and the test compounds were added to different wells at different time points before the temperature shift (-4 h and -2 h) and every 2 h after the temperature shift (0, 2, 4,…, 48 h). At 72 h post the temperature shift cells were fixed with 3.76% formaldehyde in PBS at room temperature (Sigma, F8775-500ML) for 10 minutes and then permeablized in 0.2% Triton X-100 (VWR, CA97062-208) for 2 minutes. Cells were then washed three times with 1X PBS, cover slips were blocked with 2% milk in 1X PBS, and incubated with the indicated primary mouse anti NS5A antibody (9E10) (1∶2000) at 4°C overnight. Cover slips were then washed three times with PBS-T (0.1% tween 20) and incubated with Alexa Fluor 594 donkey anti-mouse secondary antibody (Invitrogen catalogue no. A21203) for 45 min at room temperature. Cover slips were then washed three times with PBS-T (0.1% tween 20) and stained with Hoechst (1∶5000) in 1X PBS for 5 min and again washed three times with 1X PBS. Cells then were visualized using Zeiss inverted Axiovert 200 M microscope with a 20x/0.8 objective lens. The infection levels were calculated by counting infected and total cells numbers in ten random fields for each condition. The non-treated cell infection level was normalized to 100% and all other infections were calculated based on that normalization.

### MTT cell viability assay

Cells were grown in 96-well tissue culture plates and treated with HCV or peptides as described above. Cells were washed with PBS and incubated in 0.3 mg/mL MTT (3-(4,5-dimethylthiazol-2-yl)-2,5-diphenyltetrazolium bromide) (Sigma, M2128) for 1 h. Cells were then lysed with DMSO for 10 min and lysates were transferred to an optical plate. The O.D. at 570 nm for each sample was analyzed.

### Accession numbers

Kap β3/IPO5- NM_002271, Kap α1- NM_002264, Imp β1- NM_002265, HPRT- NM_000194.2, HCV JFH- HM049503, and HCV H77- JX472013.

**All other Materials and Methods are described in S1 Methods.**

## Supporting Information

S1 Figure **The binding of nuclear transport signals or HCV proteins to nuclear transport factors.** Using an ELISA assay system, we verified that the domains shown in Fig. 1 (and Table 3) are able to bind the different NTFs. ELISA plates were coated with the indicated peptides and their binding to the indicated NTFs was determined. The four NLS sequences in core were bound to (A) IPOA5 and to (B) IPO5. (C) The NS2 NLS and the second NLS of NS3 were bound to IPOA5 while NS5A NLS showed very weak binding and the first NLS of NS3 did not show any binding to IPOA5. (D) NS2 NLS did not bind to IPO5 while the two NLS sequences of NS3 and the one of NS5A bound IPO5. (E) All potential NES sequences were shown to bind to XPO1 similar to the HIV-1 Rev NES which was used as a positive control. (F) SV40 NLS, which bound only IPOA5, and HIV-1 Rev NLS, which bound specifically to IPO5, were used as positive controls. A known mutant of SV40 NLS which does not bind IPOA5 was used as a negative control. In all cases, the reverse sequence of the potential NLS did not show any binding to any NTFs inferring the binding is sequence specific. For panels G-I, plates were coated by the indicated HCV proteins and scanned for binding to (G) IPOA5; (H) IPO5; and (I) XPO1. Core (green) bound to all three NTFs; NS2 (red) bound only to IPOA5 and XPO1; NS3 bound to IPOA5 and IPO5, but not to XPO1; and NS5A bound to IPO5 alone. Based on the binding curves the apparent K_d(app)_ was calculated using GraphPad Prism software ver. 5 (see Table 3A for panels A-F and Table 3B for panels G-I). For all panels n = 3.
(TIF)

S2 Figure **BiFC combinations.** HCV proteins (blue) and NTFs (green) were conjugated by a linker (red) to half-YFP molecules (yellow and black stripes) at their N or C terminus. This figure summarizes all the possible combinations for interaction between two proteins.
(TIF)

S3 Figure **Synthetic peptides bearing putative nuclear transport signals disrupt NTFs-HCV proteins interactions in-vitro.** Plates were coated with the indicated HCV proteins and scanned for binding to IPOA5 (A-C), IPO5 (D-F), and XPO1 (G-H) in the presence of increasing concentrations of synthetic peptides bearing the different nuclear transport signal sequences. Positive control peptides were SV40 NLS, which disrupts IPOA5 interactions, HIV-1 Rev NLS, which disrupts IPO5 interactions, and HIV-1 Rev NES, which disrupts XPO1 interactions. The negative control peptides were non-active SV40 mut NLS and the known cell permeability peptide, Pen, which do not interfere with IPOA5, IPO5 or XPO1 binding. See also S1 Figure. For all panels n = 3.
(TIF)

S4 Figure **Synthetic peptides bearing putative nuclear transport signals disrupt NTFs-HCV protein interactions in cells.** Cells were transfected with plasmids encoded to the indicated HCV proteins linked to the V5 epitope and were treated with the indicated peptides at 100 µM. After 2 days of incubation the cells were lysed, and the HCV proteins (A) core, (B) NS2, (C) NS3, and (D) NS5A were immunoprecipitated with anti V5 antibody and were scanned by Western blot Co-IP of IPOA5 or IPO5. The same procedure was done with the Co-IP of (E) core and (F) NS2 with XPO1. As positive controls, we used SV40 NLS, which is known to disrupt IPOA5 interactions, and HIV-1 Rev NLS, which is known to disrupt IPO5 interactions. We also used the non-active peptide Pen peptide as a control, which is known to allow cell permeability; it was also conjugated to all the peptides used in this experiment to ensure their cell penetration. The control lane was an empty vector control.
(TIF)

S5 Figure **Schematic structure of the double GFP plasmids.** Schematic structure of the double GFP plasmid as well as the NLS/SLN and the NES/SEN bearing plasmid that were used to study the functionality of the putative nuclear transport signals.
(TIF)

S6 Figure **Functionality of the putative nuclear transport signals in cells: Quantitation.** Fluorescent intensity of the results presented in Fig. 4 was quantified using ImageJ v. 1.45s. The efficiency of the transport is summarized in Table 4.
(TIF)

S7 Figure **Functionality of the putative nuclear transport signals in cells: Reverse sequences controls.** Cells were transfected by vectors encoded to the double GFP with the different reverse nuclear transport signal sequences. At 24 h post transfection, cells were fixed and stained with Hoechst. Cells were visualized using a Zeiss inverted Axiovert 200 M microscope for localization of the GFP. In order to distinguish the nuclei, cells were stained with Hoechst. A dashed yellow square marks the magnified area. Fluorescent intensity was quantified using ImageJ v. 1.45 s. The efficiency of the transport is summarized in Table 4.
(TIF)

S8 Figure **Relocalization of NLS reporters derived from HCV proteins in HCV infected cells.** Cells were infected with HCV and transfected by the different double GFP tagged nuclear transport signal encoding plasmids 3 days PI. (A) NS3 NLS 1, (B) NS5A NLS, and (C) core NLS 1. At 24 h post transfection, cells were stained for core to monitor infection and with Hoechst to stain the nuclei.
(TIF)

S1 Table **Summary of all combinations of the BiFC.** The intensity of the florescence obtained from the BiFC study. Florescence signal was ranked as: +++ for strong, ++ for moderate, + for weak and – for no florescence.
(DOC)

S2 Table **Summary of primers and cloning conditions.**
(DOC)

S1 Methods **Extended Material and Methods.**
(DOCX)
