# Supplementary figures and images for: Functional Characterization of Nuclear Localization and Export Signals in Hepatitis C Virus Proteins and Their Role in the Membranous Web
Source: PLoS One. 2014 Dec 8;9(12):e114629. doi: 10.1371/journal.pone.0114629 (PMC4259358; doi:10.1371/journal.pone.0114629)

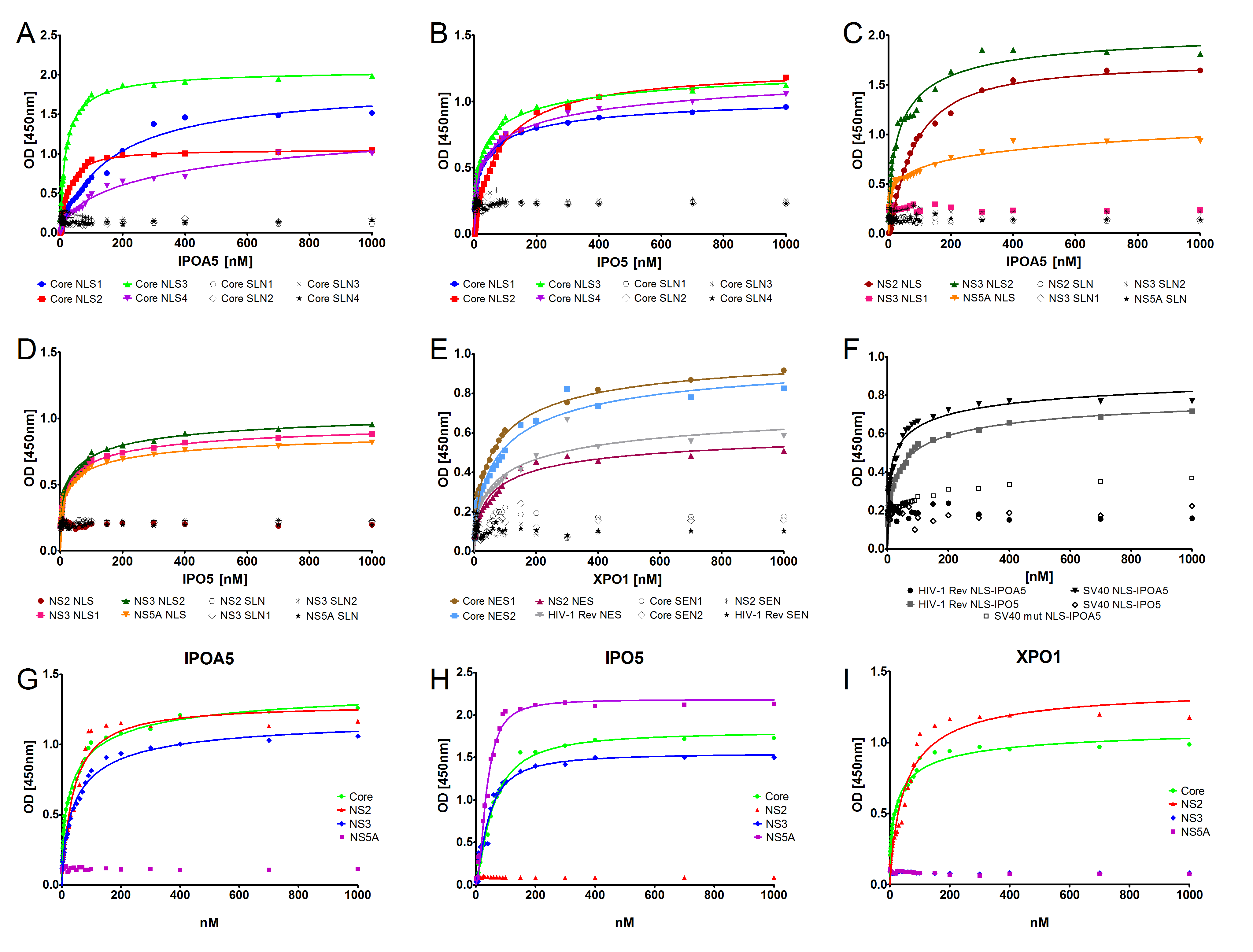

Supplement: S1 Figure — The binding of nuclear transport signals or HCV proteins to nuclear transport factors. Using an ELISA assay system, we verified that the domains shown in Fig. 1 (and Table 3) are able to bind the different NTFs. ELISA plates were coated with the indicated peptides and their binding to the indicated NTFs was determined. The four NLS sequences in core were bound to (A) IPOA5 and to (B) IPO5. (C) The NS2 NLS and the second NLS of NS3 were bound to IPOA5 while NS5A NLS showed very weak binding and the first NLS of NS3 did not show any binding to IPOA5. (D) NS2 NLS did not bind to IPO5 while the two NLS sequences of NS3 and the one of NS5A bound IPO5. (E) All potential NES sequences were shown to bind to XPO1 similar to the HIV-1 Rev NES which was used as a positive control. (F) SV40 NLS, which bound only IPOA5, and HIV-1 Rev NLS, which bound specifically to IPO5, were used as positive controls. A known mutant of SV40 NLS which does not bind IPOA5 was used as a negative control. In all cases, the reverse sequence of the potential NLS did not show any binding to any NTFs inferring the binding is sequence specific. For panels G-I, plates were coated by the indicated HCV proteins and scanned for binding to (G) IPOA5; (H) IPO5; and (I) XPO1. Core (green) bound to all three NTFs; NS2 (red) bound only to IPOA5 and XPO1; NS3 bound to IPOA5 and IPO5, but not to XPO1; and NS5A bound to IPO5 alone. Based on the binding curves the apparent Kd(app) was calculated using GraphPad Prism software ver. 5 (see Table 3A for panels A-F and Table 3B for panels G-I). For all panels n = 3. (TIF) [file pone.0114629.s001.tif]

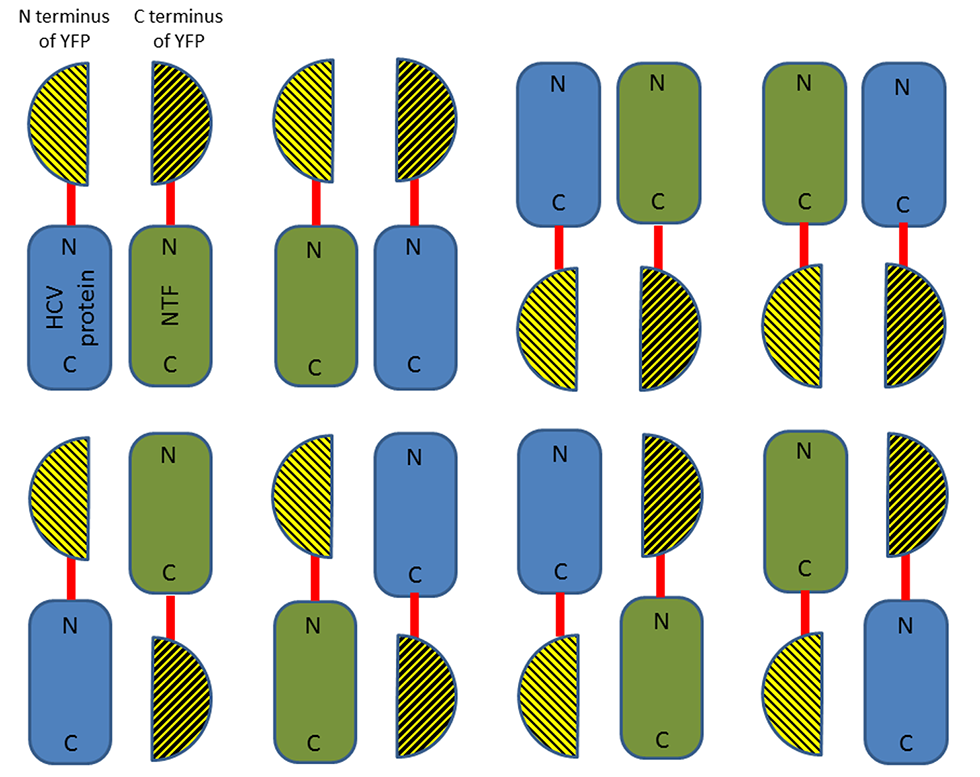

Supplement: S2 Figure — BiFC combinations. HCV proteins (blue) and NTFs (green) were conjugated by a linker (red) to half-YFP molecules (yellow and black stripes) at their N or C terminus. This figure summarizes all the possible combinations for interaction between two proteins. (TIF) [file pone.0114629.s002.tif]

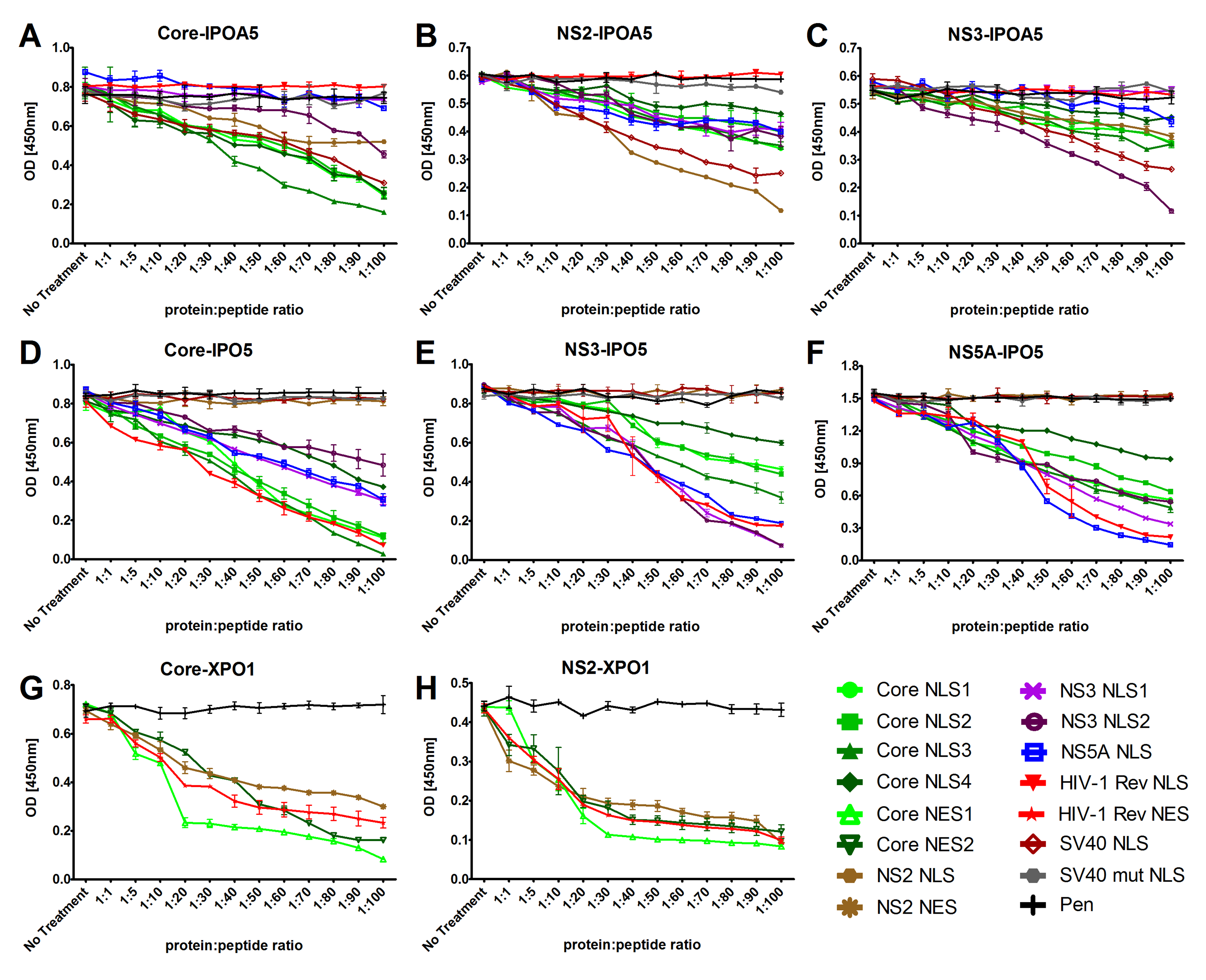

Supplement: S3 Figure — Synthetic peptides bearing putative nuclear transport signals disrupt NTFs-HCV proteins interactions in-vitro. Plates were coated with the indicated HCV proteins and scanned for binding to IPOA5 (A-C), IPO5 (D-F), and XPO1 (G-H) in the presence of increasing concentrations of synthetic peptides bearing the different nuclear transport signal sequences. Positive control peptides were SV40 NLS, which disrupts IPOA5 interactions, HIV-1 Rev NLS, which disrupts IPO5 interactions, and HIV-1 Rev NES, which disrupts XPO1 interactions. The negative control peptides were non-active SV40 mut NLS and the known cell permeability peptide, Pen, which do not interfere with IPOA5, IPO5 or XPO1 binding. See also S1 Figure. For all panels n = 3. (TIF) [file pone.0114629.s003.tif]

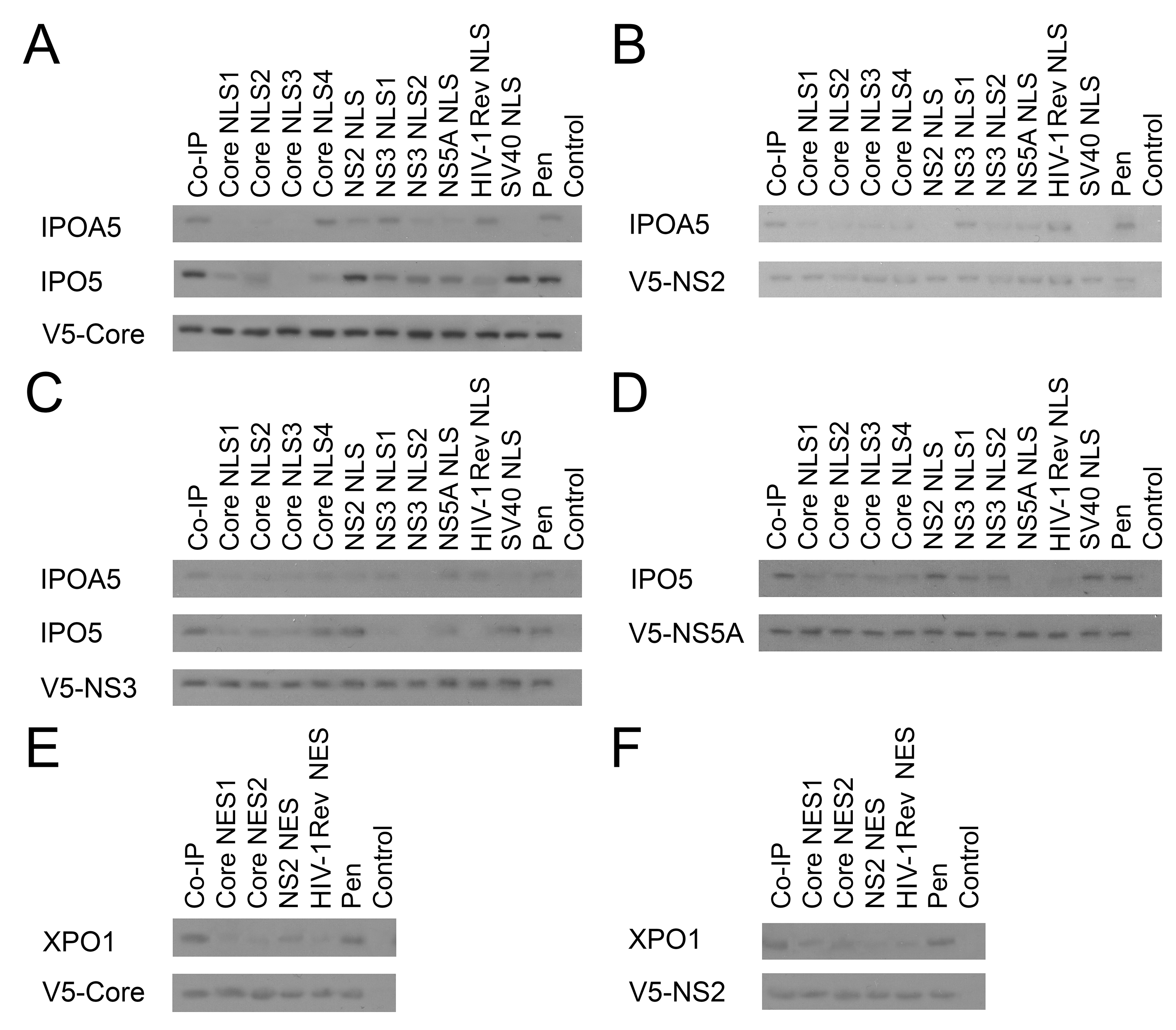

Supplement: S4 Figure — Synthetic peptides bearing putative nuclear transport signals disrupt NTFs-HCV protein interactions in cells. Cells were transfected with plasmids encoded to the indicated HCV proteins linked to the V5 epitope and were treated with the indicated peptides at 100 µM. After 2 days of incubation the cells were lysed, and the HCV proteins (A) core, (B) NS2, (C) NS3, and (D) NS5A were immunoprecipitated with anti V5 antibody and were scanned by Western blot Co-IP of IPOA5 or IPO5. The same procedure was done with the Co-IP of (E) core and (F) NS2 with XPO1. As positive controls, we used SV40 NLS, which is known to disrupt IPOA5 interactions, and HIV-1 Rev NLS, which is known to disrupt IPO5 interactions. We also used the non-active peptide Pen peptide as a control, which is known to allow cell permeability; it was also conjugated to all the peptides used in this experiment to ensure their cell penetration. The control lane was an empty vector control. (TIF) [file pone.0114629.s004.tif]

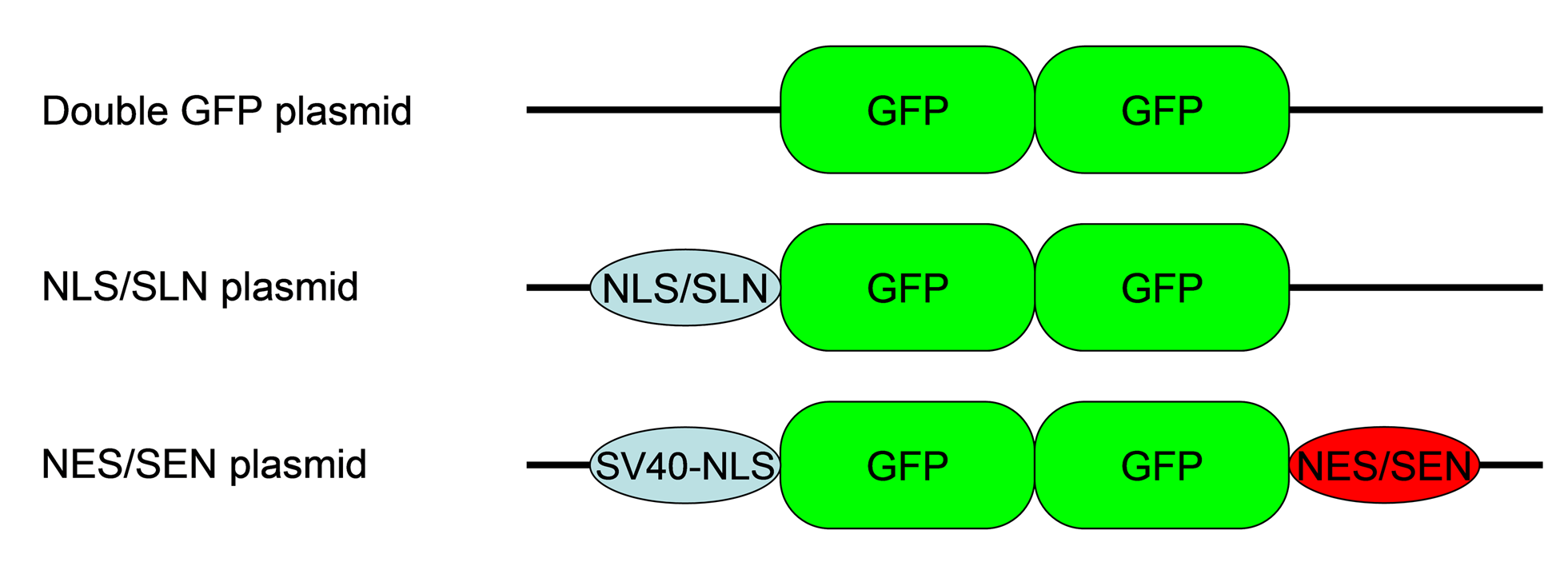

Supplement: S5 Figure — Schematic structure of the double GFP plasmids. Schematic structure of the double GFP plasmid as well as the NLS/SLN and the NES/SEN bearing plasmid that were used to study the functionality of the putative nuclear transport signals. (TIF) [file pone.0114629.s005.tif]

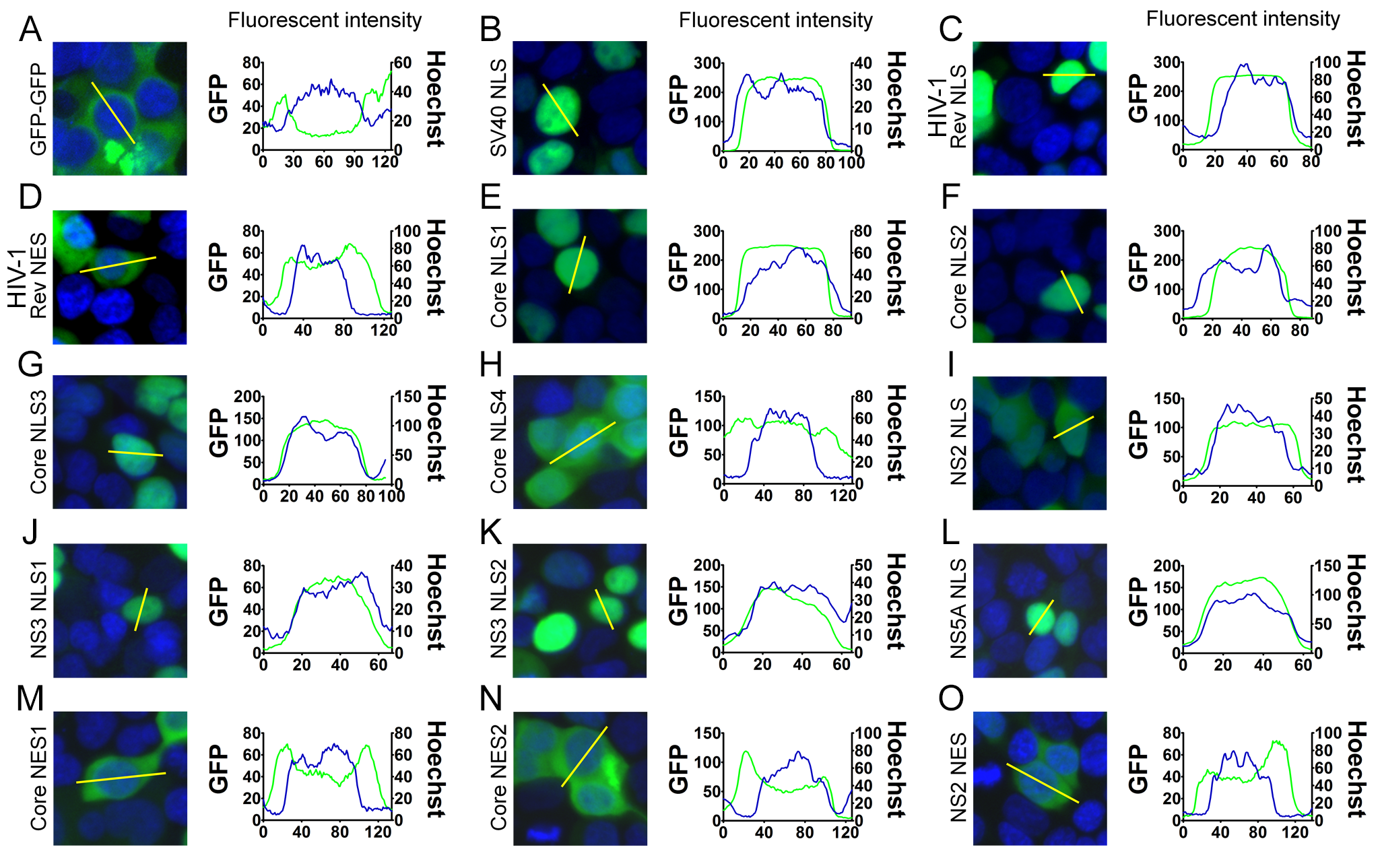

Supplement: S6 Figure — Functionality of the putative nuclear transport signals in cells: Quantitation. Fluorescent intensity of the results presented in Fig. 4 was quantified using ImageJ v. 1.45s. The efficiency of the transport is summarized in Table 4. (TIF) [file pone.0114629.s006.tif]

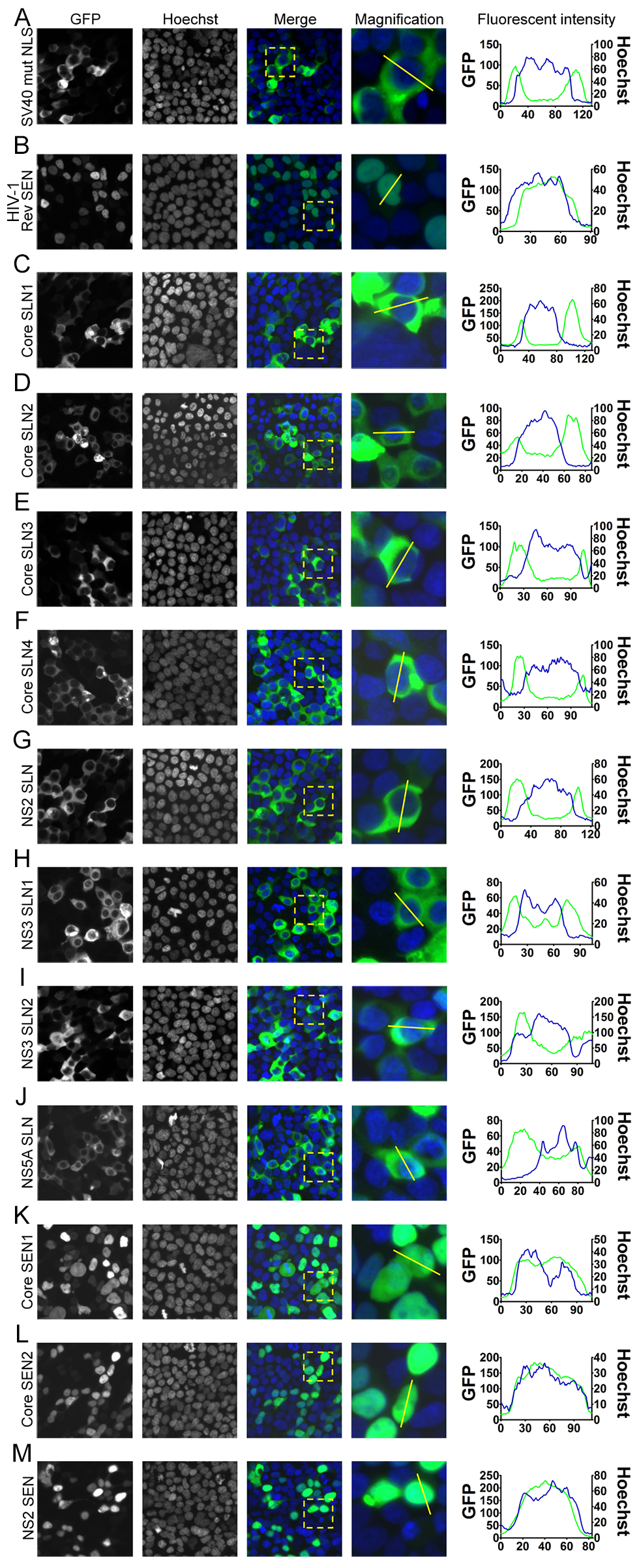

Supplement: S7 Figure — Functionality of the putative nuclear transport signals in cells: Reverse sequences controls. Cells were transfected by vectors encoded to the double GFP with the different reverse nuclear transport signal sequences. At 24 h post transfection, cells were fixed and stained with Hoechst. Cells were visualized using a Zeiss inverted Axiovert 200 M microscope for localization of the GFP. In order to distinguish the nuclei, cells were stained with Hoechst. A dashed yellow square marks the magnified area. Fluorescent intensity was quantified using ImageJ v. 1.45 s. The efficiency of the transport is summarized in Table 4. (TIF) [file pone.0114629.s007.tif]

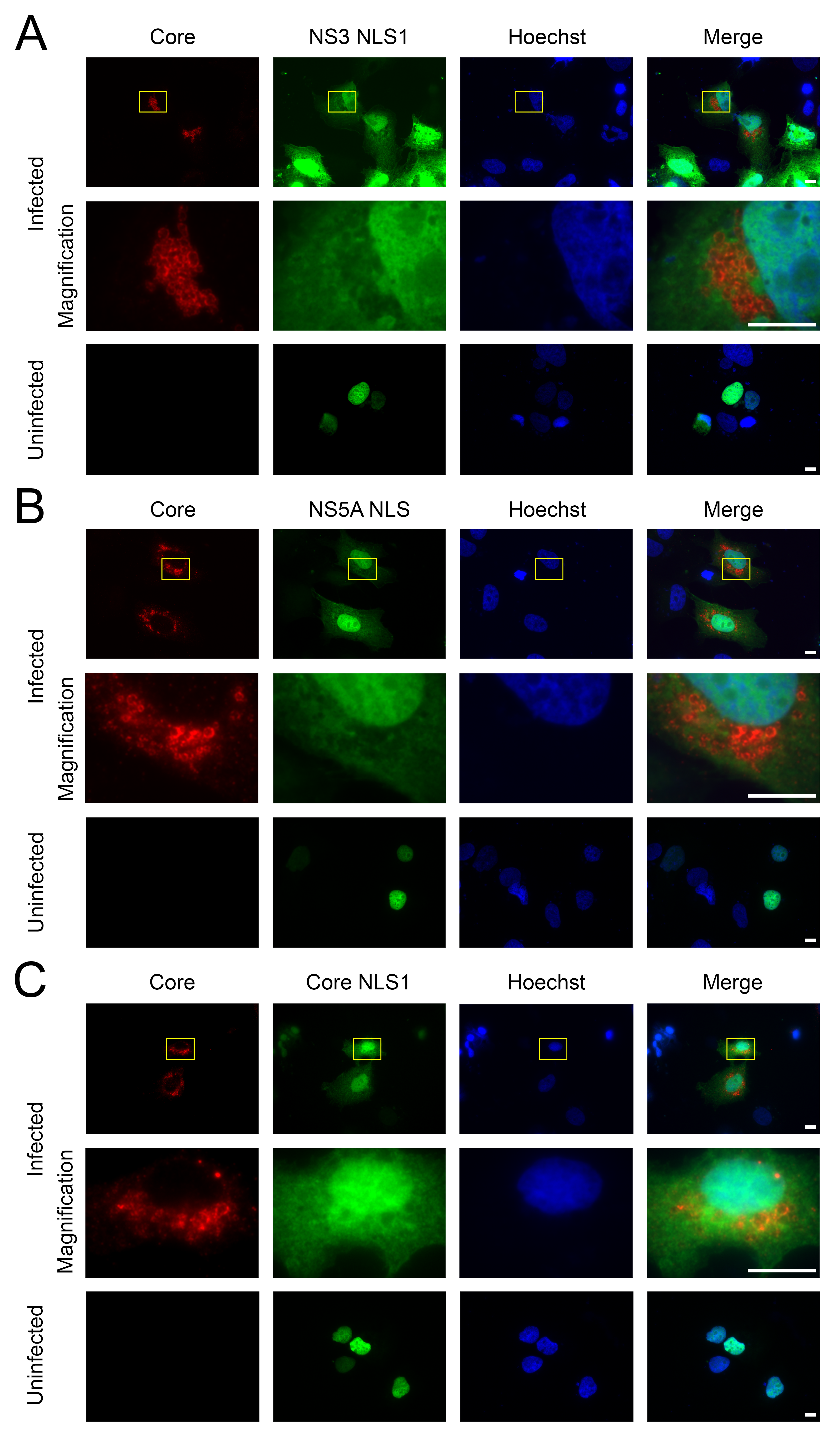

Supplement: S8 Figure — Relocalization of NLS reporters derived from HCV proteins in HCV infected cells. Cells were infected with HCV and transfected by the different double GFP tagged nuclear transport signal encoding plasmids 3 days PI. (A) NS3 NLS 1, (B) NS5A NLS, and (C) core NLS 1. At 24 h post transfection, cells were stained for core to monitor infection and with Hoechst to stain the nuclei. (TIF) [file pone.0114629.s008.tif]
