# Supplementary material for: Functional Characterization of Nuclear Localization and Export Signals in Hepatitis C Virus Proteins and Their Role in the Membranous Web
Source: PLoS One. 2014 Dec 8;9(12):e114629. doi: 10.1371/journal.pone.0114629 (PMC4259358; doi:10.1371/journal.pone.0114629)
Supplement: S1 Table — Summary of all combinations of the BiFC. The intensity of the florescence obtained from the BiFC study. Florescence signal was ranked as: +++ for strong, ++ for moderate, + for weak and – for no florescence. (DOC) [file pone.0114629.s009.doc]

Table S1: **Summary of all combinations of the BiFC.**

|  | **IPOA5 -YC** | **YC- IPOA5** | **IPOA5 -YN** | **YN- IPOA5** | **IPO5 -YC** | **YC- IPO5** | **IPO5 -YN** | **YN- IPO5** | **XPO1 -YC** | **YC- XPO1** | **XPO1 -YN** | **YN- XPO1** | **Linker -YC** | **YC- Linker** | **Linker -YN** | **YN- Linker** |
| --- | --- | --- | --- | --- | --- | --- | --- | --- | --- | --- | --- | --- | --- | --- | --- | --- |
| **Core-YN** | **+++** | **+++** |  |  | **++** | **+++** |  |  | **+++** | **+++** |  |  | **-** | **-** |  |  |
| **Core-YC** |  |  | **++** | **++** |  |  | **+++** | **+++** |  |  | **+++** | **+** |  |  | **-** | **-** |
| **YN-Core** | **+++** | **++** |  |  | **++** | **++** |  |  | **+** | **<1%** |  |  | **-** | **-** |  |  |
| **YC-Core** |  |  | **++** | **++** |  |  | **++** | **++** |  |  | **+** | **<1%** |  |  | **-** | **-** |
| **NS2-YN** | **++** | **++** |  |  | **-** | **-** |  |  | **+** | **+** |  |  | **-** | **-** |  |  |
| **NS2-YC** |  |  | **+++** | **++** |  |  | **-** | **-** |  |  | **<1%** | **<1%** |  |  | **-** | **-** |
| **YN-NS2** | **++** | **++** |  |  | **-** | **-** |  |  | **++** | **++** |  |  | **-** | **-** |  |  |
| **YC-NS2** |  |  | **++** | **+** |  |  | **-** | **-** |  |  | **+++** | **+** |  |  | **-** | **-** |
| **NS3-YN** | **++** | **++** |  |  | **++** | **+++** |  |  | **-** | **-** |  |  | **-** | **-** |  |  |
| **NS3-YC** |  |  | **++** | **+** |  |  | **++** | **+++** |  |  | **-** | **-** |  |  | **-** | **-** |
| **YN-NS3** | **+** | **+** |  |  | **+** | **+++** |  |  | **-** | **-** |  |  | **-** | **-** |  |  |
| **YC-NS3** |  |  | **+++** | **+++** |  |  | **+++** | **++** |  |  | **-** | **-** |  |  | **-** | **-** |
| **NS4-YN** | **-** | **-** |  |  | **-** | **-** |  |  | **-** | **-** |  |  | **-** | **-** |  |  |
| **NS4-YC** |  |  | **-** | **-** |  |  | **-** | **-** |  |  | **-** | **-** |  |  | **-** | **-** |
| **YN-NS4** | **-** | **-** |  |  | **-** | **-** |  |  | **-** | **-** |  |  | **-** | **-** |  |  |
| **YC-NS4** |  |  | **-** | **-** |  |  | **-** | **-** |  |  | **-** | **-** |  |  | **-** | **-** |
| **NS5A-YN** | **-** | **-** |  |  | **++** | **++** |  |  | **-** | **-** |  |  | **-** | **-** |  |  |
| **NS5A-YC** |  |  | **-** | **-** |  |  | **++** | **++** |  |  | **-** | **-** |  |  | **-** | **-** |
| **YN-NS5A** | **-** | **-** |  |  | **++** | **+** |  |  | **-** | **-** |  |  | **-** | **-** |  |  |
| **YC-NS5A** |  |  | **-** | **-** |  |  | **++** | **+++** |  |  | **-** | **-** |  |  | **-** | **-** |
| **Linker-YN** | **-** | **-** |  |  | **-** | **-** |  |  | **-** | **-** |  |  | **-** | **-** |  |  |
| **Linker-YC** |  |  | **-** | **-** |  |  | **-** | **-** |  |  | **-** | **-** |  |  | **-** | **-** |
| **YN-Linker** | **-** | **-** |  |  | **-** | **-** |  |  | **-** | **-** |  |  | **-** | **-** |  |  |
| **YC-Linker** |  |  | **-** | **-** |  |  | **-** | **-** |  |  | **-** | **-** |  |  | **-** | **-** |
