# Supplementary material for: Functional Characterization of Nuclear Localization and Export Signals in Hepatitis C Virus Proteins and Their Role in the Membranous Web
Source: PLoS One. 2014 Dec 8;9(12):e114629. doi: 10.1371/journal.pone.0114629 (PMC4259358; doi:10.1371/journal.pone.0114629)
Supplement: S2 Table — Summary of primers and cloning conditions. (DOC) [file pone.0114629.s010.doc]

**Table S2A: Primers and cloning information for V5-tagged HCV protein vectors.**

| Name | Sequence | PCR conditions | Taq polymerase |
| --- | --- | --- | --- |
| Core gate-F | AGTTTGTACAAAAAAGCAGGCTAAATGAGCACGAATCCTAAACCTCAAAG | 94ºC 1min | Platinum Taq DNA Polymerase |
| Core gate-R | CACTTTGTACAAGAAAGCTGGGTTGGCTGAAGCGGGCACAGTCAGG | 30 cycles of: 94ºC 30sec, 62ºC 30sec, 72ºC 1min | (Invitrogen 10966-034) |
|  |  | 72ºC 10min |  |
| NS2 gate-F | AGTTTGTACAAAAAAGCAGGCTAACTGGACACGGAGGTGGCCGCG | 94ºC 1min | Platinum Taq DNA Polymerase |
| NS2 gate-R | CACTTTGTACAAGAAAGCTGGGTTCAGCAACCTCCACCCCTTGGAG | 30 cycles of: 94ºC 30sec, 67ºC 30sec, 72ºC 1min |  |
|  |  | 72ºC 10min |  |
| NS3 gate-F | AGTTTGTACAAAAAAGCAGGCTAAGCGCCCATCACGGCGTACGCC | 94ºC 2min | Platinum Taq DNA Polymerase |
| NS3 gate-R | CACTTTGTACAAGAAAGCTGGGTTCGTGACGACCTCCAGGTCGGCC | 30 cycles of: 94ºC 30sec, 68ºC 30sec, 72ºC 2.5min |  |
|  |  | 72ºC 10min |  |
| NS4a gate-F | AGTTTGTACAAAAAAGCAGGCTAAAGCACCTGGGTGCTCGTTGGCG | 94ºC 30sec | Platinum Taq DNA Polymerase |
| NS4a gate-R | CACTTTGTACAAGAAAGCTGGGTTGCACTCTTCCATCTCATCGAACTC | 30 cycles of: 94ºC 30sec, 64ºC 30sec, 72ºC 1min |  |
|  |  | 72ºC 10min |  |
| NS5a gate-F | AGTTTGTACAAAAAAGCAGGCTAATCCGGTTCCTGGCTAAGGGACAT | 94ºC 2min | Platinum Taq DNA Polymerase |
| NS5a gate-R | CACTTTGTACAAGAAAGCTGGGTTGCAGCACACGACATCTTCCGTGT | 30 cycles of: 94ºC 30sec, 65ºC 30sec, 72ºC 2.5min |  |
|  |  | 72ºC 10min |  |

**Table S2B: Primers and cloning information for HIS tagged E. coli expression vectors.**

| Name | Sequence | Restriction enzymes | PCR conditions | Taq polymerase |
| --- | --- | --- | --- | --- |
| Core-exp-F | ATATGAGCTCATGAGCACGAATCCTAAACCTCAAAG | SacI (Invitrogen 15240-201) | 94ºC 1min | Platinum Taq DNA Polymerase |
| Core-exp-R | ATTTCTTAAGTTAGGCTGAAGCGGGCACAGTCAGG | AflII (NEB R0520L) | 30 cycles of: 94ºC 30sec, 58ºC 30sec, 72ºC 1min |  |
|  |  |  | 72ºC 10min |  |
| NS2-exp-F | ATATGAGCTCCTGGACACGGAGGTGGCCGCG | SacI | 94ºC 1min | Platinum Taq DNA Polymerase |
| NS2-exp-R | TAAACTCGAGCAGCAACCTCCACCCCTTGGAGA | XhoI (Invitrogen 15231-012) | 30 cycles of: 94ºC 30sec, 65ºC 30sec, 72ºC 1min |  |
|  |  |  | 72ºC 10min |  |
| NS3-exp-F | ATATCTTAAGTCCCCGGTGTTCACGGACAACT | AflII | 94ºC 2min | KOD Xtreme™ |
| NS3 exp-R | ATTTCGATCGCGTGACGACCTCCAGGTC | PvuI (NEB R0150L) | 30 cycles of: 98ºC 10sec, 62ºC 30sec, 68ºC 2.5min | Hot Start DNA Polymerase |
|  |  |  | 68ºC 10min | (Novagen 71975) |
| NS5A-exp-F | ATATCGCCGGCGTCCGGTTCCTGGCTAAGG | MreI (Fermentas ER2021) | 94ºC 2min | KOD Xtreme™ |
| NS5A-exp-R | TAAACTCGAGCTTGAGAGATGGAGCGGACAGCT | XhoI | 30 cycles of: 98ºC 10sec, 63ºC 30sec, 68ºC 2.5min | Hot Start DNA Polymerase |
|  |  |  | 68ºC 10min |  |
| IPOA5-exp-F | ATATGAGCTCATGACCACCCCAGGAAAAGAG | SacI | 94ºC 2min | Platinum Taq High Fidelity |
| IPOA5-exp-R | ATATCTTAAGTTAAAGCTGGAAACCTTCCATAGGA | AflII | 30 cycles of: 94ºC 30sec, 54ºC 30sec, 68ºC 3min | (Invitrogen 11304-011) |
|  |  |  | 68ºC 10min |  |
| IPO5-exp-F | AAATCGCCGGCGATGGCGGCGGCCGC | NaeI (NEB R0190L) | 94ºC 2min | KOD Xtreme™ |
| IPO5-exp-R | ACCTCTCGAGTCACGCAGAGTTCAGGAGCTCCT | XhoI | 30 cycles of: 98ºC 10sec, 68ºC 3min | Hot Start DNA Polymerase |
|  |  |  | 68ºC 10min |  |
| XPO1-exp-F | AAATCGCCGGCGATGCCAGCAATTATGA | NaeI | 94ºC 2min | Platinum Taq High Fidelity |
| XPO1-exp-R | ACCTCTCGAGTTAATCACACATTTCTTCTGGAATCTC | XhoI | 30 cycles of: 94ºC 30sec, 58ºC 30sec, 68ºC 3min |  |
|  |  |  | 68ºC 10min |  |

exp - expression

**Table S2C: Primers and cloning information for mammalian BiFC expression vectors.**

| Name | Sequence | Restriction enzymes | PCR conditions | Taq polymerase |
| --- | --- | --- | --- | --- |
| YC/YN-link-coreF | ATATCTAGAATGAGCACGAATCCTAAACCTCAAAG | XbaI (Invitrogen 15226-012) | 94ºC 1min | Platinum Taq DNA |
| YC/YN-link-coreR | ATATCTAGATTAGGCTGAAGCGGGCACAGTCAGG | XbaI | 30 cycles of: 94ºC 30sec, 55ºC 30sec, 72ºC 1min | Polymerase |
|  |  |  | 72ºC 10min |  |
| Link-YN/YC-coreF | ATAAAGCTTATGAGCACGAATCCTAAACCTCAAAG | HindIII (Invitrogen 15207-038) | 94ºC 1min | Platinum Taq DNA |
| Link-YN/YC-coreR | AATAGGATCCGGCTGAAGCGGGCACAGTCAGG | BamHI (NEB R0136M) | 30 cycles of: 94ºC 30sec, 55ºC 30sec, 72ºC 1min | Polymerase |
|  |  |  | 72ºC 10min |  |
| YC/YN-link-NS2F | ATACTCGAGCTGGACACGGAGGTGGCCGCG | XhoI | 94ºC 1min | Platinum Taq DNA |
| YC/YN-link-NS2R | ATATCTAGATTACAGCAACCTCCACCCCTTGGAG | XbaI | 30 cycles of: 94ºC 30sec, 60ºC 30sec, 72ºC 1min | Polymerase |
|  |  |  | 72ºC 10min |  |
| Link-YN/YC-NS2F | ATAAAGCTTATGCTGGACACGGAGGTGGCCGCG | HindIII | 94ºC 1min | Platinum Taq DNA |
| Link-YN/YC-NS2R | AATAGGATCCCAGCAACCTCCACCCCTTGGAG | BamHI | 30 cycles of: 94ºC 30sec, 65ºC 30sec, 72ºC 1min | Polymerase |
|  |  |  | 72ºC 10min |  |
| YC/YN-link-NS3F | ATATCTAGAGCGCCCATCACGGCGTACGCC | XbaI | 94ºC 2min | Platinum Taq High Fidelity |
| YC/YN-link-NS3R | ATATCTAGATTACGTGACGACCTCCAGGTCGGCC | XbaI | 30 cycles of: 94ºC 30sec, 64ºC 30sec, 68ºC 3min |  |
|  |  |  | 68ºC 10min |  |
| Link-YN/YC-NS3F | ATAAAGCTTATGGCGCCCATCACGGCGTACGCC | HindIII | 94ºC 2min | Platinum Taq High Fidelity |
| Link-YN/YC-NS3R | AATAGGATCCCGTGACGACCTCCAGGTCGGCC | BamHI | 30 cycles of: 94ºC 30sec, 65ºC 30sec, 68ºC 3min |  |
|  |  |  | 68ºC 10min |  |
| YC/YN-link-NS4AF | ATACTCGAGAGCACCTGGGTGCTCGTTGGCG | XhoI | 94ºC 30sec | Platinum Taq DNA |
| YC/YN-link-NS4AR | ATATCTAGATTAGCACTCTTCCATCTCATCGAACTC | XbaI | 30 cycles of: 94ºC 30sec, 56ºC 30sec, 72ºC 1min | Polymerase |
|  |  |  | 72ºC 10min |  |
| Link-YN/YC-NS4AF | ATAAAGCTTATGAGCACCTGGGTGCTCGTTGGCG | HindIII | 94ºC 30sec | Platinum Taq DNA |
| Link-YN/YC-NS4AR | AATAGGATCCGCACTCTTCCATCTCATCGAACTC | BamHI | 30 cycles of: 94ºC 30sec, 60ºC 30sec, 72ºC 1min | Polymerase |
|  |  |  | 72ºC 10min |  |
| YC/YN-link-NS5AF | ATACTCGAGTCCGGTTCCTGGCTAAGGGACAT | XhoI | 94ºC 2min | Platinum Taq High Fidelity |
| YC/YN-link-NS5AR | ATATCTAGATTAGCAGCACACGACATCTTCCGTGT | XbaI | 30 cycles of: 94ºC 30sec, 59ºC 30sec, 68ºC 3min |  |
|  |  |  | 68ºC 10min |  |

| Name | Sequence | Restriction enzymes | PCR conditions | Taq polymerase |
| --- | --- | --- | --- | --- |
| Link-YN/YC-NS5AF | ATAAAGCTTATGTCCGGTTCCTGGCTAAGGGACAT | HindIII | 94ºC 2min | Platinum Taq High Fidelity |
| Link-YN/YC-NS5AR | AATAGGTACCGCAGCACACGACATCTTCCGTGT | KpnI (NEB R0142M) | 30 cycles of: 94ºC 30sec, 61ºC 30sec, 68ºC 3min |  |
|  |  |  | 68ºC 10min |  |
| YC/YN-Link-IPOA5F | AAATCTCGAGATGACCACCCCAGGAAAAGAG | XhoI | 94ºC 2min | Platinum Taq High Fidelity |
| YC/YN-Link-IPOA5R | ATATTCTAGATTAAAGCTGGAAACCTTCCATAGGA | XbaI | 30 cycles of: 94ºC 30sec, 56ºC 30sec, 68ºC 3min |  |
|  |  |  | 68ºC 10min |  |
| Link-YN/YC-IPOA5F | AAATGCTAGCATGACCACCCCAGGAAAAGAG | NheI (NEB R0131L) | 94ºC 2min | Platinum Taq High Fidelity |
| Link-YN/YC-IPOA5R | TATAGGATCCAAGCTGGAAACCTTCCATAGGA | BamHI | 30 cycles of: 94ºC 30sec, 59ºC 30sec, 68ºC 3min |  |
|  |  |  | 68ºC 10min |  |
| YC/YN-link-IPO5F | AATACTCGAGATGGCGGCGGCCGCGGC | XhoI | 94ºC 2min | Platinum Taq High Fidelity |
| YC/YN-link-IPO5R | ATACTCGAGTCACGCAGAGTTCAGGAGCTCCT | XhoI | 30 cycles of: 94ºC 30sec, 65ºC 30sec, 68ºC 3min |  |
|  |  |  | 68ºC 10min |  |
| Link-YN/YC-IPO5F | ATAGGTACCATGGCGGCGGCCGCGGCGG | KpnI | 94ºC 2min | Platinum Taq High Fidelity |
| Link-YN/YC-IPO5R | AATAGGTACCCGCAGAGTTCAGGAGCTCCTGAA | KpnI | 30 cycles of: 94ºC 30sec, 65ºC 30sec, 68ºC 3min |  |
|  |  |  | 68ºC 10min |  |
| YC/YN-Link-XPO1F | AAATCTCGAGATGCCAGCAATTATGACAATG | XhoI | 94ºC 2min | Platinum Taq High Fidelity |
| YC/YN-Link-XPO1R | ATATTCTAGATTAATCACACATTTCTTCTGGAATCTC | XbaI | 30 cycles of: 94ºC 30sec, 52ºC 30sec, 68ºC 3min |  |
|  |  |  | 68ºC 10min |  |
| Link-YN/YC-XPO1F | AAATGCTAGCATGCCAGCAATTATGACAATG | NheI | 94ºC 2min | Platinum Taq High Fidelity |
| Link-YN/YC-XPO1R | TATAGGATCCATCACACATTTCTTCTGGAATCTC | BamHI | 30 cycles of: 94ºC 30sec, 55ºC 30sec, 68ºC 3min |  |
|  |  |  | 68ºC 10min |  |

Table S2D: **Primers and cloning information for nuclear transport signal cloning to double GFP vectors.**

| Name | Sequence | Restriction enzymes | PCR conditions | Taq polymerase |
| --- | --- | --- | --- | --- |
| Core NLS1-F | AATAGCTAGCATGAGCACGAATCCTAAACCTCA | NheI | 94ºC 2min | KOD Xtreme™ |
| Core NLS1-R | TAAACTTAAGCTTGACGTCCTGTGGGCG | AflII | 30 cycles of: 98ºC 10sec, 60ºC 30sec, 68ºC 30sec | Hot Start DNA Polymerase |
|  |  |  | 68ºC 10min |  |
| Core NLS2-F | AATAGCTAGCATGGGAGTTTACTTGTTGCCGC | NheI | 94ºC 2min | KOD Xtreme™ |
| Core NLS2-R | TAAACTTAAGGCGCACACCCAATCTAGG | AflII | 30 cycles of: 98ºC 10sec, 58ºC 30sec, 68ºC 30sec | Hot Start DNA Polymerase |
|  |  |  | 68ºC 10min |  |
| Core NLS3-F | AATAGCTAGCATGCAACCTCGAGGTAGACGTCAG | NheI | 94ºC 2min | KOD Xtreme™ |
| Core NLS3-R | TAAACTTAAGGGGCTGAGCCCAGGTC | AflII | 30 cycles of: 98ºC 10sec, 60ºC 30sec, 68ºC 30sec | Hot Start DNA Polymerase |
|  |  |  | 68ºC 10min |  |
| Core NLS4-F | AATAGCTAGCATGCCTAGCTGGGGCC | NheI | 94ºC 2min | KOD Xtreme™ |
| Core NLS4-R | TAAACTTAAGCTTACCCAAATTGCGCGA | AflII | 30 cycles of: 98ºC 10sec, 57ºC 30sec, 68ºC 30sec | Hot Start DNA Polymerase |
|  |  |  | 68ºC 10min |  |
| Core NES1-F | ATATCTAGAGTCATCGATACCCTTACGTGC | XbaI | 94ºC 2min | KOD Xtreme™ |
| Core NES1-R | AATGGGCCCTTACCCCATGAGGTCGGC | ApaI (NEB R0114L) | 30 cycles of: 98ºC 10sec, 56ºC 30sec, 68ºC 30sec | Hot Start DNA Polymerase |
|  |  |  | 68ºC 10min |  |
| Core NES2-F | ATATCTAGACTTCCTGGTTGCTCTTTCTCTATC | XbaI | 94ºC 2min | KOD Xtreme™ |
| Core NES2-R | AATGGGCCCTTAGGCTGAAGCGGGCAC | ApaI | 30 cycles of: 98ºC 10sec, 56ºC 30sec, 68ºC 30sec | Hot Start DNA Polymerase |
|  |  |  | 68ºC 10min |  |
| NS2 NLS-F | AATAGCTAGCATGGCTGTGGAACCAGTCGTCTTCTC | NheI | 94ºC 2min | KOD Xtreme™ |
| NS2 NLS-R | TAAACTTAAGGGTATCTGCCCCCCACGTGATG | AflII | 30 cycles of: 98ºC 10sec, 61ºC 30sec, 68ºC 30sec | Hot Start DNA Polymerase |
|  |  |  | 68ºC 10min |  |
| NS2 NES-F | ATATCTAGATATTACAAGCGCTACATCAGCTGGT | XbaI | 94ºC 2min | KOD Xtreme™ |
| NS2 NES-R | AATGGGCCCTTATTCTACTCTGGTCAGAAAATACTGAA | ApaI | 30 cycles of: 98ºC 10sec, 58ºC 30sec, 68ºC 30sec | Hot Start DNA Polymerase |
|  |  |  | 68ºC 10min |  |
| NS3 NLS1-F | AATAGCTAGCATGCTTTACCTGGTCACGAGGCAC | NheI | 94ºC 2min | KOD Xtreme™ |
| NS3 NLS1-R | TAAACTTAAGTTTCAAGTAGGAAATGGGCCGGG | AflII | 30 cycles of: 98ºC 10sec, 60ºC 30sec, 68ºC 30sec | Hot Start DNA Polymerase |
|  |  |  | 68ºC 10min |  |
| Name | Sequence | Restriction enzymes | PCR conditions | Taq polymerase |
| NS3 NLS2-F | AATAGCTAGCATGACAACCACGCTCCCCCAG | NheI | 94ºC 2min | KOD Xtreme™ |
| NS3 NLS2-R | TAAACTTAAGTGCCACAAATCTGTAGATGCCTGG | AflII | 30 cycles of: 98ºC 10sec, 59ºC 30sec, 68ºC 30sec | Hot Start DNA Polymerase |
|  |  |  | 68ºC 10min |  |
| NS5A NLS-F | AATAGCTAGCATGAAATCCCCTCCTGTGCCTC | NheI | 94ºC 2min | KOD Xtreme™ |
| NS5A NLS-R | TAAACTTAAGGAGGACCACCGTACGACG | AflII | 30 cycles of: 98ºC 10sec, 59ºC 30sec, 68ºC 30sec | Hot Start DNA Polymerase |
|  |  |  | 68ºC 10min |  |
| HIV-1 Rev NLS-F | AATAGCTAGCATGAGACAGGCTCGAAGGAATAGAA | NheI | 94ºC 2min | KOD Xtreme™ |
| HIV-1 Rev NLS-R | TAAACTTAAGTCTCTGTCTCTCTCTCCACCTTC | AflII | 30 cycles of: 98ºC 10sec, 58ºC 30sec, 68ºC 30sec | Hot Start DNA Polymerase |
|  |  |  | 68ºC 10min |  |
| HIV-1 Rev NES-F | ATATCTAGACCTGTGCCTCTTCAGCTACC | XbaI | 94ºC 2min | KOD Xtreme™ |
| HIV-1 Rev NES-R | AATGGGCCCTTAGTTACAATCAAGAGTAAGTCTCTCAAGC | ApaI | 30 cycles of: 98ºC 10sec, 59ºC 30sec, 68ºC 30sec | Hot Start DNA Polymerase |
|  |  |  | 68ºC 10min |  |

**Table S2E: Oligos and cloning information for reverse nuclear transport signal cloning to double GFP vectors.**

| Name | Sequence | Restriction enzymes |
| --- | --- | --- |
| Core SLN1 | AATAGCTAGCATGAAGGTCGACCAGCCACGCCGTAACACCAACCGTAAAACCAAAAGACAACCTAAACCTAATACGAGCATGCTTAAGTTTA | NheI and AflII |
| Core SLN1c | TTATCGATCGTACTTCCAGCTGGTCGGTGCGGCATTGTGGTTGGCATTTTGGTTTTCTGTTGGATTTGGATTATGCTCGTACGAATTCAAAT |  |
| Core SLN2 | AATAGCTAGCATGCGCGTGGGTTTGAGACCTGGCAGGCGCCCGTTGTTGTACGTTGGACTTAAGTTTA | NheI and AflII |
| Core SLN2c | TTATCGATCGTACGCGCACCCAAACTCTGGACCGTCCGCGGGCAACAACATGCAACCTGAATTCAAAT |  |
| Core SLN3 | AATAGCTAGCATGCCCCAGGCTTGGACCAGGGGCGAGCCCCGGCGTGCAAAGCCCATCCCTCAGCGTAGAGGTCGACCTCAACTTAAGTTTA | NheI and AflII |
| Core SLN3c | TTATCGATCGTACGGGGTCCGAACCTGGTCCCCGCTCGGGGCCGCACGTTTCGGGTAGGGAGTCGCATCTCCAGCTGGAGTTGAATTCAAAT |  |
| Core SLN4 | AATAGCTAGCATGAAGGGTTTGAATCGCTCGAGGCGTCGGCCCGACACACCCGGCTGGAGCCCTCTTAAGTTTA | NheI and AflII |
| Core SLN4c | TTATCGATCGTACTTCCCAAACTTAGCGAGCTCCGCAGCCGGGCTGTGTGGGCCGACCTCGGGAGAATTCAAAT |  |
| Core SEN1 | ATATCTAGAGGGATGCTCGACGCCTTCGGCTGCACGCTTACCGATATCGTCTAAGGGCCCATT | XbaI and ApaI |
| Core SEN1c | TATAGATCTCCCTACGAGCTGCGGAAGCCGACGTGCGAATGGCTATAGCAGATTCCCGGGTAA |  |
| Core SEN2 | ATATCTAGAGCCTCAGCTCCCGTGACTCTGTGCTCTCTCCTGGCCCTGCTTTTCATCTCTTTCTCTTGCGGTCCTCTTTAAGGGCCCATT | XbaI and ApaI |
| Core SEN2c | TATAGATCTCGGAGTCGAGGGCACTGAGACACGAGAGAGGACCGGGACGAAAAGTAGAGAAAGAGAACGCCAGGAGAAATTCCCGGGTAA |  |
| NS2 SLN | AATAGCTAGCATGGGGTGGACGATCCTCAAGACCGAGATGCGATCCTTCGTCGTCCCACTTAAGTTTA | NheI and AflII |
| NS2 SLNc | TTATCGATCGTACCCCACCTGCTAGGAGTTCTGGCTCTACGCTAGGAAGCAGCAGGGTGAATTCAAAT |  |
| NS2 SEN | ATATCTAGAACCCTGTTTTATCAGCTTTGGTGGATGTGCTGGAGCATCTACCGCAAGTACTATCCATCGCTGTAAGGGCCCATT | XbaI and ApaI |
| NS2 SENc | TATAGATCTTGGGACAAAATAGTCGAAACCACCTACACGACCTCGTAGATGGCGTTCATGATAGGTAGCGACATTCCCGGGTAA |  |
| NS3 SLN1 | AATAGCTAGCATGCCCTCGCTTCTGAGCGGTAGGAGCGATGGTCGACGGCGCGTGCCCATTGTCGATGCCCACAGGACGGTCCTTAAGTTTA | NheI and AflII |
| NS3 SLN1c | TTATCGATCGTACGGGAGCGAAGACTCGCCATCCTCGCTACCAGCTGCCGCGCACGGGTAACAGCTACGGGTGTCCTGCCAGGGATTCAAAT |  |
| NS3 SLN2 | AATAGCTAGCATGTACATCGGCCCAAAGGGGAGGGGCACTAGGGGCCGGCGCCAAACTAGGTCCGTCGCTGATCAGCCCCTCCTTAAGTTTA | NheI and AflII |
| NS3 SLN2c | TTATCGATCGTACATGTAGCCGGGTTTCCCCTCCCCGTGATCCCCGGCCGCGGTTTGATCCAGGCAGCGACTAGTCGGGGAGGAATTCAAAT |  |
| NS5A SLN | AATAGCTAGCATGCTCGTCGTGACGCGTCGTCGGCGGCCTCCGCCTGTGCCTCCTTCCAAACTTAAGTTTA | NheI and AflII |
| NS5A SLNc | TTATCGATCGTACGAGCAGCACTGCGCAGCAGCCGCCGGAGGCGGACACGGAGGAAGGTTTGAATTCAAAT |  |
| HIV-1 Rev SLN | AATAGCTAGCATGAGACAGAGAGAGAGATGGAGGAGAAGAAGAAATAGGCGAGCTCAGAGACTTAAGTTTA | NheI and AflII |
| HIV-1 Rev SLNc | TTATCGATCGTACTCTGTCTCTCTCTCTACCTCCTCTTCTTCTTTATCCGCTCGAGTCTCTGAATTCAAAT |  |
| HIV-1 Rev SEN | ATATCTAGACTTACTCTTAGAGAGCTTCCGCCACTACAGCTTTAAGGGCCCATT | XbaI and ApaI |
| HIV-1 Rev SENc | TATAGATCTGAATGAGAATCTCTCGAAGGCGGTGATGTCGAAATTCCCGGGTAA |  |
| SV40 mut | AATAGCTAGCATGCCAAAGACTAAGCGCAAGGTACTTAAGTTTA | NheI and AflII |
| SV40 mutc | TTATCGATCGTACGGTTTCTGATTCGCGTTCCATGAATTCAAAT |  |

Table S2F: **Real time PCR primers and probes.**

| Name | Sequence |
| --- | --- |
| HCV forward | TCTGCGGAACCGGTGAGTA |
| HCV revers | GTGTTTCTTTTGGTTTTTCTTTGAGGTTTAGG |
| HCV FAM probe | FAM-CACGGTCTACGAGACCTCCCGGGGCAC-TAMARA |
| HPRT Syber F | CCTGGCGTCGTGATTAGTG |
| HPRT Syber R | ACACCCTTTCCAAATCCTCAG |
