# Supplementary material for: Functional Characterization of Nuclear Localization and Export Signals in Hepatitis C Virus Proteins and Their Role in the Membranous Web
Source: PLoS One. 2014 Dec 8;9(12):e114629. doi: 10.1371/journal.pone.0114629 (PMC4259358; doi:10.1371/journal.pone.0114629)
Supplement: S1 Methods — Extended Material and Methods. (DOCX) [file pone.0114629.s011.docx]

**Extended Materials and Methods**

**Constructs**

*Mammalian expression vectors V5 tagged*

Constructs encoding for HCV proteins were created by cloning H77 strain sequences into gateway entry vector pDONR201 (Invitrogen, 11798-014) by recombination using gateway BP clonase II (Invitrogen, 11789-020). Sequences were then transferred to pcDNA3.1/nV5-DEST gateway vector (Invitrogen, 12290010) by recombination using gateway LR clonase II (Invitrogen, 11791-020). HCV sequences included core (amino acids 1-192), NS2 (amino acids 811-1027), NS3 (amino acids 1028-1658), NS4A (amino acids 1659-1712) and NS5A (amino acids 1974-2421). Primers, and PCR conditions are presented in Table S2A. All other conditions were according to the manufacturer’s protocol.

*E. coli expression vectors*

Cloning of the HCV and NTFs into pET-21 expression vectors was done by common cloning methods. The pcDNA3.1/nV5-DEST with the different HCV genes (mentioned above) were used as the cloning template for the HCV proteins; the template for IPOA5 was a Myc-DDK-tagged ORF clone of IPOA5 (ORIGENE RC210333); the template for IPO5 was a pGEX4T-3 vector containing the IPO5 ORF a gift from Nabeel R. Yaseen (Cornell University Medical College) [1]; and the template for XPO1 was a Myc-DDK-tagged ORF clone of XPO1 (ORIGENE RC206004). Primers, restriction enzymes and PCR conditions are presented in Table S2B. All other conditions were according to the manufacturer’s protocol. All ligations were done using T4 DNA Ligase.

*Bi-fluorescent complementation (BiFC) vectors*

Cloning of the HCV and NTFs into BiFC vectors [2,3] was done by common cloning methods. BiFC vectors were received from Tom Kerppola (University of Michigan Medical School). The pcDNA3.1/nV5-DEST with the different HCV genes (mentioned above) were used as the template for the cloning of the HCV proteins; The template for IPOA5 was a Myc-DDK-tagged ORF clone of IPOA5; The template for IPO5 was a pGEX4T-3 vector encoding to GST-IPO5; and the template for XPO1 was a Myc-DDK-tagged ORF clone of XPO1. Each gene was cloned to four vectors; YC-Linker (containing the C terminal sequence of YFP to generate YC-Linker-Gene vector); YN-Linker (containing the N terminal sequence of YFP to generate YN-Linker-Gene vector); Linker-YC (containing the C terminal sequence of YFP to generate Gene-Linker-YC vector); and Linker-YN (containing the N terminal sequence of YFP to generate Gene-Linker-YN vector). Primers, restriction enzymes and PCR conditions are shown in Table S2C. All other conditions were according to the manufacturer’s protocol. All ligations were done using T4 DNA Ligase.

*Double GFP tagged reporter vectors*

Cloning of the HCV NLS sequences into a double GFP vectors and the NES sequences to SV40 NLS double GFP vectors was done by common cloning methods. Both the double GFP and SV40 NLS double GFP were generous gifts from Fabio Beltram (Scuola Normale Superiore and Italian Institute of Technology, Pisa, Italy) [4]. The pcDNA3.1/nV5-DEST with the different HCV genes (mentioned above) were used as the cloning templates for the HCV NTFs; HIV-1 Rev-GFP plasmid [5] was used as the cloning template for Rev NLS and NES. Primers, restriction enzymes and PCR conditions are shown in Table S2D. All other conditions were according to the manufacturer’s protocol. All ligations were done using T4 DNA Ligase.

For the reverse sequences as well as for the SV40 mut sequence, oligos containing the DNA sequence to code for the reverse sequence (SLN for SEN) and their complement sequences were ordered from IDT (Coralville, Iowa USA) (Table S2E). The ordered sequences included restriction sites to allow specific ligation to the double GFP vector. Each pair of complementing sequences were mixed and denatured at 98ºC for 10 min. The mixture was cooled slowly to room temperature to allow annealing of the two sequences. Following the annealing step the double stranded oligos were cut using the indicated restriction enzymes and ligated to the double GFP vectors (Table S2E). All other conditions were according to the manufacturer’s protocol. All ligations were done using T4 DNA Ligase.

**Protein expression and purification**

*Core*

Expression and purification of full length core were performed according to methods described in a previous publication [6] with the following modifications: *Escherichia coli* BL21(DE3) transformed with the pET21-core were cultured in 5ml LB medium containing 100 µg/ml of ampicillin (LB-Amp) overnight at 37ºC and 220 RPM; then, the culture was diluted 1:100 in 100ml LB-Amp and grown overnight at 37ºC and 220 RPM. The culture was then diluted 1:10 in 1L LB-Amp at 37ºC and 220 RPM. Protein expression was induced when the OD_600nm_ reached approximately 0.8 by addition of 1 mM Isopropyl-β-D-thiogalactopyranoside (IPTG) and the culture incubated for 20 h at 25ºC and 200 RPM. The culture was then centrifuged at 4ºC for 30 min at 6000 x g. The cell pellet from a 1L culture was resuspended in 40 ml of 20 mM Tris-HCl (pH 8.0) and sonicated on ice. After centrifugation at 4ºC for 20 min at 18000 x g, the cell pellet containing inclusion bodies was resuspended in 15 ml of UNT buffer (20 mM Tris-HCl, pH 8.0, 2 M urea, 0.5 M NaCl, 2% Triton X-100), and sonicated and centrifuged again as described above. After washing the pellet with UNT buffer and UNT buffer lacking urea, the proteins in the pellet fraction were denatured in 25 ml of buffer A (20 mM Tris-HCl, pH 8.0, 0.5 M NaCl, 5 mM imidazole, 6 M guanidine hydrochloride, 1 mM β-mercaptoethanol) by stirring for 4 h at 4ºC. After centrifugation at 18000 x g for 20 min at 4ºC, the supernatant was applied to a Ni-NTA-agarose (Qiagen, Hilden, Germany) column pre-equilibrated with buffer A at 4ºC. After washing with buffer A, the column was further washed with washing buffer (20 mM Tris-HCl, pH 8.0, 0.5 M NaCl, 20 mM imidazole, 1 mM β-mercaptoethanol) containing 6 M urea. The core protein bound to the column was then refolded with a linear gradient of guanidine hydrochloride (6–0 M) in washing buffer, and eluted with a 250mM imidazole in washing buffer (without guanidine hydrochloride). All washing and elution was done at 4ºC. Fractions containing the core protein were pooled and dialyzed against a storage buffer (50 mM Tris-HCl, pH 8.0, 150 mM NaCl, 1 mM DTT, 10% glycerol) and aliquots were stored at -80ºC.

*NS2*

Expression and purification of full length NS2 were performed according to methods similar to those used for core with several modifications: *Escherichia coli* BL21(DE3) transformed with the pET21-NS2 were cultured in 5ml LB medium containing 100 µg/ml of ampicillin (LB-Amp) overnight at 37ºC and 220 RPM; then, the culture was diluted 1:100 in 100ml LB-Amp and grown overnight at 37ºC and 220 RPM. The culture was then diluted 1:10 in 1L LB-Amp and incubated at 37ºC and 220 RPM. Protein expression was induced when the OD_600nm_ reached approximately 0.6 by addition of 1 mM IPTG and the culture was incubated at 30ºC and 180 RPM overnight. The culture was then centrifuged at 4ºC for 30 min at 8000 x g. The cell pellet from a 1L culture was resuspended in 40 ml of 25mM Hepes, pH 7.6, 0.3M NaCl, 20% glycerol, 10mM β-mercaptoethanol, and 0.1% CHAPS and sonicated on ice. After centrifugation at 4ºC for 20 min at 22000 x g, the cell pellet containing inclusion bodies was resuspended in 15 ml of buffer A (50mM Hepes, pH 7.6, 1M NaCl, 20% glycerol, 10mM β-mercaptoethanol, 0.1% CHAPS, 2 M urea), and sonicated and centrifuged as described above. After washing the pellet with buffer A and buffer A lacking urea, proteins in the pellet fraction were denatured in 25 ml of buffer B (50mM Hepes, pH 7.6, 1M NaCl, 20% glycerol, 10mM β-mercaptoethanol, 0.1% CHAPS, 6M guanidine hydrochloride) by stirring for 4 h at 4ºC. After centrifugation at 22000 x g for 20 min at 4ºC, the supernatant was applied to a Ni-NTA-agarose (Qiagen, Hilden, Germany) column pre-equilibrated with buffer A at 4ºC. After washing with buffer B, the column was further washed with washing buffer B containing 20 mM imidazole. The NS2 protein bound to the column was then refolded with a linear gradient of guanidine hydrochloride (6–0 M) in washing buffer (50mM Hepes, pH 7.6, 0.5M NaCl, 20% glycerol, 10mM β-mercaptoethanol, 0.1% CHAPS, 20 mM imidazole), and eluted with a 250mM imidazole in washing buffer (without guanidine hydrochloride). All washing and elution was performed at 4ºC. Fractions containing the NS2 protein were pooled and dialyzed against a storage buffer (50mM Hepes, pH 7.6, 150M NaCl, 20% glycerol, 10mM β-mercaptoethanol, 0.1% CHAPS) and aliquots were stored at -80ºC.

*NS3*

Expression and purification of full length NS3 were pperformed according to methods described in a previous publication [7] with the following modifications: *Escherichia coli* BL21(DE3) transformed with the pET21-NS3 were cultured in 5ml LB-Amp overnight at 37ºC and 220 RPM; then, the culture was diluted 1:100 in 100ml LB-Amp and grown overnight at 37ºC and 220 RPM. The culture was then diluted 1:10 in 1L LB-Amp and incubated at 37ºC and 220 RPM. Protein expression was induced when the OD_600nm_ reached approximately 0.6 by addition of 1 mM IPTG and the culture was incubated at 30ºC and 150 RPM overnight. The culture was then centrifuged at 4ºC for 30 min at 6000 x g. All purification steps were performed at 4ºC. The culture pellet was resuspended in 30ml of 25mM Hepes, pH 7.6, 0.3M NaCl, 20% glycerol, 10mM β-mercaptoethanol, and 0.1% CHAPS and sonicated on ice. After centrifugation at 4ºC for 40 min at 25000 x g, the supernatant was applied to an Ni-NTA-agarose (Qiagen, Hilden, Germany) column pre-equilibrated with buffer A (50mM Hepes, pH 7.6, 1M NaCl, 20% glycerol, 10mM β-mercaptoethanol, and 0.1% CHAPS at 4ºC). The column was washed with buffer A containing 50mM imidazole. The His-tagged HCV-NS3 protein was eluted with buffer A containing 250mM imidazole. Fractions containing the NS3 protein were pooled and aliquots were stored at -80ºC.

*NS5A*

Expression and purification of full length NS5A were performed according to methods described in a previous publication [8] with the following modifications: *Escherichia coli* BL21(DE3) transformed with the pET21-NS5A were cultured in 5ml LB-Amp overnight at 37ºC and 220 RPM; then, culture was diluted 1:100 in 100ml LB-Amp and grown overnight at 37ºC and 220 RPM. The culture was then diluted 1:10 in 1L LB-Amp at 37ºC and 220 RPM. Protein expression was induced when the OD_600nm_ reached approximately 1 by addition of 0.5 mM IPTG and the culture wad incubated at 20ºC for 14 h and at 150 RPM. The culture was then centrifuged at 4ºC for 20 min at 8000 x g. All purification steps were performed at 4ºC. The culture pellet was resuspended in 20ml of lysis buffer (100mM Tris, pH 8.0, 200mM NaCl, and 10mM β- mercaptoethanol 1% Triton X-100,with protease inhibitors pepstatin A (7.5 µg/ml) and leupeptin (7.5 µg/ml) and 1mM PMSF) and sonicated on ice. After centrifugation for at 4ºC for 30 min at 25000 x g, the supernatant was applied to a Ni-NTA-agarose (Qiagen, Hilden, Germany) column pre-equilibrated with lysis buffer. The column was washed with lysis buffer containing 10mM imidazole and then with lysis buffer contain 20mM imidazole. The His-tagged HCV-NS5A protein was eluted with lysis buffer containing 250mM imidazole. Fractions containing the NS5 protein were pooled and dialyzed against 50mM Hepes, pH 7.5, 1mM DTT overnight. Glycerol was added to a final concentration of 20% after dialysis. Sample aliquots were stored at -80ºC.

*IPOA5, IPO5*

Expression and purification of full length IPOA5, IPO5 were perform according to methods described previously [9] with the following modification: *Escherichia coli* BL21(DE3) transformed with the pET21- IPOA5 or IPO5 were cultured in 5ml LB-Amp overnight at 37ºC and 220 RPM; then, the culture was diluted 1:100 in 100ml LB-Amp and grown overnight at 37ºC and 220 RPM. The culture was then diluted 1:10 in 1L LB-Amp and incubated at 37ºC and 220 RPM. Protein expression was induced when the OD_600nm_ reached approximately 0.5 by the addition of 1 mM IPTG and the culture was incubated at 25ºC for 4h at 150 RPM. The culture was then centrifuged at 4ºC for 30 min at 6000 x g and frozen at -80 ºC overnight. All purification steps were performed at 4ºC. The culture pellet was resuspended in 40ml of ice-cold lysis buffer (50 mM Tris–HCl (pH 8.0), 100 mM NaCl, 10% glycerol, 0.1% Triton X-100, 0.1 mM PMSF and 0.1 mM benzamidine) and sonicated on ice. After centrifugation at 4ºC for 20 min at 15000 x g, the supernatant was applied to an Ni-NTA-agarose (Qiagen, Hilden, Germany) column pre-equilibrated with buffer A (50 mM Tris–HCl (pH 8.0), 150 mM NaCl, 10% glycerol, 0.1% Triton X-100) at 4ºC. The column was washed with buffer A containing 25mM imidazole. The His-tagged IPOA5 or IPO5 protein was eluted with buffer A containing 250mM imidazole. Fractions containing the eluted protein were pooled and dialyzed against a storage buffer (PBS containing 10% glycerol) and aliquots were stored at -80ºC.

*XPO1*

Expression and purification of full length XPO1 were perform according to methods described in a previous publication [10] with the following modification: *Escherichia coli* BL21(DE3) transformed with the pET21-XPO1 were cultured in 5ml LB-Amp overnight at 37ºC and 220 RPM; then, the culture was diluted 1:100 in 100ml LB-Amp and grown overnight at 37ºC and 220 RPM. The culture was then diluted 1:10 in 1L LB-Amp and incubated at 37ºC and 220 RPM. Protein expression was induced when the OD_600nm_ reached approximately 0.8 by addition of 1 mM IPTG and the culture was incubated at 30ºC for 8 h and at 150 RPM. The culture was then centrifuged at 4ºC for 20 min at 6000 x g. All purification steps were performed at 4ºC. The culture pellet was resuspended in 30ml of ice cold lysis buffer (200 mm Tris HCl, pH 7.5, 0.5mM NaCl, 5 mM β-mercaptoethanol Triton X-100, 1mM PMSF) and sonicated on ice. After centrifugation for at 4ºC for 30 min at 18000 x g, the supernatant was applied to a Ni-NTA-agarose (Qiagen, Hilden, Germany) column pre-equilibrated with lysis buffer. The column was washed with lysis buffer containing 10mM imidazole and then with lysis buffer contain 20mM imidazole. The His-tagged HCV-XPO1 protein was eluted with buffer containing 250mM imidazole. Fractions containing the XPO1 protein were pooled and dialyzed against 20 mM HEPES KOH, pH 7.5, 80 mM CH_3_COOK, 4 mM (CH_3_COO)_2_Mg, 250 mM sucrose and 1mM DTT overnight. Sample aliquots were stored at -80ºC.

**Localization of nuclear transport signal-double GFP reporter**

*In HEK293T cells*

Nuclear transport signal-double GFP constructs were transfected into HEK293T cells using lipofectamine 2000 reagent (Invitrogen, 11668019). At 24 h post transfection cells were fixed with 3.76% formaldehyde at room temperature (Sigma, F8775-500ML) for 8 min then permeabilized in 0.2% Triton X-100 (VWR, CA97062-208) for 2 min. Cells were washed three times with X1 PBS and were stained with Hoechst 1:5000 in X1 PBS for 5 min followed by three washes with X1 PBS. The cells were visualized using a Zeiss inverted Axiovert 200M microscope with a 20x/0.8 objective lens to examine localization of the GFP. At least ten random fields of cells were imaged for each combination. Channels were merged using ImageJ software (National Institutes of Health). Photoshop Elements 10 (Adobe) software was used to adjust brightness and contrast levels for individual images and to assemble images into figures.

*In Huh7.5 cells*

The localization study in Huh7.5 cells was performed as described above with the following modifications. The constructs were transfected into infected or uninfected Huh7.5 cells using lipofectamine 2000 reagent in a reverse transfection. Briefly, the Huh7.5 cells were trypsinized and added to the transfection mixture on a caver slip. Post fixation and permeabilization, the cover slips were incubated with the anti HCV core primary antibodies (Thermo Scientific catalogue no. MA1-080) at 4°C overnight. Samples were then washed three times with PBS-T (0.1% tween 20) and incubated with Alexa Fluor 594 donkey anti-mouse secondary antibody (Invitrogen catalogue no. A21203) for 45 min at room temperature. Samples were then washed three times with PBS-T (0.1% tween 20) followed by the Hoechst staining. Finally cells were visualized using an Axio Observer Z1 microscope (Carl Zeiss, Inc.) with a 63x/1.40 NA Oil UPlanS-Apochromat objective lens (Carl Zeiss Inc.). Channels were merged using ImageJ software (National Institutes of Health). Photoshop Elements 10 (Adobe) software was used to adjust brightness and contrast levels for individual images and to assemble images into figures.

**Supplementary References**

1. Yaseen NR, Blobel G (1997) Cloning and characterization of human karyopherin beta3. Proc Natl Acad Sci U S A 94: 4451-4456.

2. Hu CD, Chinenov Y, Kerppola TK (2002) Visualization of interactions among bZIP and Rel family proteins in living cells using bimolecular fluorescence complementation. Mol Cell 9: 789-798.

3. Hu CD, Kerppola TK (2003) Simultaneous visualization of multiple protein interactions in living cells using multicolor fluorescence complementation analysis. Nat Biotechnol 21: 539-545.

4. Cardarelli F, Serresi M, Bizzarri R, Giacca M, Beltram F (2007) In vivo study of HIV-1 Tat arginine-rich motif unveils its transport properties. Molecular therapy : the journal of the American Society of Gene Therapy 15: 1313-1322.

5. Levin A, Hayouka Z, Friedler A, Loyter A (2010) Over-expression of the HIV-1 Rev promotes death of nondividing eukaryotic cells. Virus Genes 40: 341-346.

6. Kang SM, Shin MJ, Kim JH, Oh JW (2005) Proteomic profiling of cellular proteins interacting with the hepatitis C virus core protein. Proteomics 5: 2227-2237.

7. Poliakov A, Hubatsch I, Shuman CF, Stenberg G, Danielson UH (2002) Expression and purification of recombinant full-length NS3 protease-helicase from a new variant of Hepatitis C virus. Protein Expr Purif 25: 363-371.

8. Huang L, Sineva EV, Hargittai MR, Sharma SD, Suthar M, et al. (2004) Purification and characterization of hepatitis C virus non-structural protein 5A expressed in Escherichia coli. Protein Expr Purif 37: 144-153.

9. Armon-Omer A, Graessmann A, Loyter A (2004) A synthetic peptide bearing the HIV-1 integrase 161-173 amino acid residues mediates active nuclear import and binding to importin alpha: characterization of a functional nuclear localization signal. J Mol Biol 336: 1117-1128.

10. Askjaer P, Jensen TH, Nilsson J, Englmeier L, Kjems J (1998) The specificity of the CRM1-Rev nuclear export signal interaction is mediated by RanGTP. J Biol Chem 273: 33414-33422.
